# Meta‐Analysis: The Placebo Response in Modern Treatments for Cholestatic Pruritus: A Systematic Review and Meta‐Analysis of Randomized Controlled Trials

**DOI:** 10.1111/apt.70915

**Published:** 2026-09-24

**Authors:** Igor Boechat Silveira, Leonardo Corrêa Süffert, Gustavo André Pedral Diniz Leite, Milena Ramos Tomé, Sarah Quick Lourenço de Lima, Gabriel André Pedral Diniz Leite, Bernardo de Faria Moraes, Pedro Robson Costa Passos, Guilherme Grossi Lopes Cançado

**Affiliations:** ^1^ Universidade Federal de Juiz de Fora Juiz de Fora Brazil; ^2^ Pontifícia Universidade Católica Do Rio Grande Do Sul Porto Alegre Brazil; ^3^ Universidade Federal de Minas Gerais Belo Horizonte Brazil; ^4^ Universidade Federal de Campina Grande Campina Grande Brazil; ^5^ Universidade Federal Do Ceará Fortaleza Brazil; ^6^ Hospital das Clínicas da Universidade Federal de Minas Gerais Belo Horizonte Brazil; ^7^ Hospital da Polícia Militar de Minas Gerais Belo Horizonte Brazil

**Keywords:** IBAT, itching, PFIC, PPAR, primary biliary cholangitis, primary sclerosing cholangitis

## Abstract

**Background/Aims:**

Cholestatic pruritus is a debilitating symptom with substantial placebo responses. We aimed to quantify this response and identify trial‐level moderators in randomized controlled trials of contemporary therapies.

**Methods:**

We performed a systematic review and meta‐analysis of placebo‐controlled trials evaluating ileal bile acid transporter inhibitors or PPAR agonists (searched through March 2026, with a supplementary search on 11 July 2026). Primary outcomes included the standardized mean change (SMC) of pruritus intensity, placebo response rate and mean difference (MD) in Numerical Rating Scale (NRS) and Worst–Itch NRS (WI‐NRS) scores. Secondary outcomes included PBC‐40 and 5‐D Itch scores. We used random‐effects models with Hartung–Knapp adjustment and meta‐regression.

**Results:**

Thirteen trials (*n* = 548) were analysed. Across placebo groups, pruritus intensity decreased significantly from baseline during follow‐up periods of up to 52 weeks. The pooled SMC was −0.49 (95% CI, −0.72 to −0.27) overall, and −0.57 (95% CI, −0.81 to −0.33) for moderate‐to‐severe pruritus. Pooled response rates were 30.0% (overall) and 27.4% (moderate‐to‐severe). In moderate‐to‐severe patients, NRS showed a significant mean reduction of −1.62, whereas the WI‐NRS change (−0.65) was not statistically significant. Significant reductions occurred in the PBC‐40 itch domain (−2.02) and overall score (−9.13), while 5‐D Itch changes were non‐significant. None of the evaluated moderators were statistically significant.

**Conclusions:**

The magnitude of placebo‐associated improvement differed across outcome instruments and may affect estimates and interpretation of treatment effects in cholestatic‐pruritus trials. These findings may inform endpoint selection and assumptions used in future trial design.

**Trial Registration:**

PROSPERO registration number: CRD420261385974

## Graphical abstract

Placebo arms show substantial improvement in cholestatic pruritus, with up to 30% response rate. Estimates differed across outcome instruments, highlighting the importance of endpoint selection and realistic placebo assumptions in future trial design.

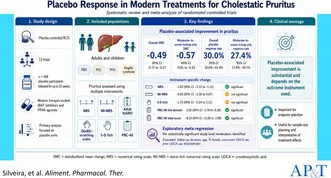

**Abbreviations**
ALP: alkaline phosphatase
ALT: alanine aminotransferase
AST: aspartate aminotransferase
CENTRAL: Cochrane Central Register of Controlled Trials
CI: confidence interval
GGT: gamma‐glutamyl transferase
GRADE: Grading of Recommendations Assessment, Development, and Evaluation
HK: Hartung–Knapp
HRQOL: health‐related quality of life
IBAT: ileal bile acid transporter
ItchRO: itch‐reported outcome measure
MD: mean difference
NRS: Numerical Rating Scale
ObsRO: observer‐reported outcome
PASI: Psoriasis Area and Severity Index
PBC: primary biliary cholangitis
PFIC: progressive familial intrahepatic cholestasis
PPAR: peroxisome proliferator‐activated receptor
PRISMA: Preferred Reporting Items for Systematic Reviews and Meta‐Analyses
PROSPERO: International Prospective Register of Systematic Reviews
PSC: primary sclerosing cholangitis
RCT: randomized controlled trial
REML: restricted maximum likelihood
RoB 2: Cochrane Risk of Bias tool
SD: standard deviation
SMC: standardized mean change
TB: total bilirubin
UDCA: ursodeoxycholic acid
VAS: Visual Analogue Scale
WI‐NRS: Worst–Itch Numerical Rating Scale

## 1 Introduction

Cholestatic pruritus is a severe, often debilitating itching sensation that imposes a significant physical, psychological and quality‐of‐life burden on patients with cholestatic liver diseases [1]. Its pathogenesis is complex and incompletely understood, involving hepatobiliary, neural and systemic mechanisms. Several mediators have been implicated, including bile acids, lysophosphatidic acid, autotaxin activity, endogenous opioids, cytokines and other pruritogens, although no single pathway fully explains the onset, severity or fluctuation of symptoms [2, 3]. This condition is a hallmark of several cholestatic liver diseases, affecting both adults and children, as seen in primary biliary cholangitis (PBC), Primary Sclerosing Cholangitis (PSC), progressive familial intrahepatic cholestasis (PFIC) and Alagille syndrome [4, 5, 6]. Despite recent therapeutic advances, including ileal bile acid transporter (IBAT) inhibitors and peroxisome proliferator‐activated receptor (PPAR) agonists, many patients continue to experience persistent or recurrent debilitating symptoms [7, 8, 9].

Accurate interpretation of treatment effects in cholestatic pruritus trials is particularly challenging because pruritus is subjective, multidimensional and inherently variable over time. In this context, the placebo response may be substantial and can directly influence trial outcomes. High placebo responses may compromise the ability of a trial to distinguish an effective intervention from an inactive control [10]. In conditions characterized by subjective and fluctuating symptoms like pruritus, this effect is often magnified by regression toward the mean or the Hawthorne effect, where natural symptom variation or the psychological impact of clinical observation leads to perceived improvement [10]. Therefore, quantifying and understanding placebo response is essential for estimating the net therapeutic benefit of emerging treatments and for optimizing the design of future randomized controlled trials in this field.

Importantly, the placebo response should not be viewed solely as a source of bias or a methodological inconvenience. Evidence from randomized controlled trials across multiple symptom‐based conditions supports placebo response as a robust psychobiological phenomenon with distinct neurobiological correlates [11]. This response is shaped by the psychosocial context of treatment and is largely mediated through mechanisms such as expectation, conditioning, patient–clinician interaction and prior therapeutic experience [11]. These mechanisms may be particularly relevant in pruritus, where symptom perception is closely linked to attention, affective state, sleep disruption, disease severity and quality of life. Although placebo response has been systematically evaluated in other gastrointestinal disorders characterized by subjective symptoms, such as in irritable bowel syndrome [12, 13], its magnitude and determinants in trials for cholestatic pruritus remain poorly defined.

Therefore, we performed a systematic review and meta‐analysis of randomized controlled trials evaluating contemporary therapies for cholestatic pruritus. Our objectives were to estimate the pooled placebo response and to explore potential trial‐level moderators that may influence its magnitude. By characterizing placebo response in this setting, we aim to provide a methodological framework to improve interpretation of antipruritic efficacy and inform the design of future clinical trials in cholestatic liver diseases.

## 2 Methods

### 2.1 Protocol Registration

This systematic review and meta‐analysis was conducted and reported in accordance with the Preferred Reporting Items for Systematic Reviews and Meta‐Analyses guidelines (PRISMA 2020) [14]. The completed PRISMA checklist is provided in Table S1. The study protocol was prospectively registered in the International Prospective Register of Systematic Reviews (PROSPERO).

### 2.2 Search Strategy and Study Selection

PubMed, Embase and CENTRAL were searched from inception to 23 March 2026, without language restrictions. In response to reviewer feedback, we conducted a supplementary search from inception to 11 July 2026, combining controlled vocabulary and free‐text terms for cholestatic liver diseases, pruritus and placebo, without requiring individual drug names. Details of the search approach and results are outlined in Table S2.

Two independent reviewers (I.B.S. and Ga.A.P.D.L.) screened titles and abstracts using Rayyan [15]. Disagreements were resolved by consensus or consultation with a senior reviewer (G.G.L.C.) when necessary. Data extraction was performed by one author (M.R.T.) using a standardized extraction form and independently verified by a second author (Gu.A.P.D.L.). When outcome data were reported only graphically, numerical values were extracted from published figures using WebPlotDigitizer [16].

### 2.3 Eligibility Criteria

Studies were eligible if they were randomized controlled trials (RCTs) enrolling patients of any age with cholestatic pruritus associated with a confirmed cholestatic liver disease, including PBC, PSC, PFIC or Alagille syndrome. Eligible trials evaluated contemporary targeted therapies, including ileal bile acid transporter (IBAT) inhibitors, such as linerixibat, maralixibat or odevixibat, or peroxisome proliferator‐activated receptor (PPAR) agonists, such as seladelpar, elafibranor or bezafibrate, compared with a matched pharmacological placebo. Only full‐text peer‐reviewed articles were eligible for inclusion; conference abstracts were excluded. A minimum follow‐up duration of 2 weeks was required. For crossover trials, only data from the first treatment period, before crossover, were included to minimize potential carryover effects.

### 2.4 Outcomes

#### 2.4.1 Primary Outcomes

This study evaluated three primary outcomes: the standardized mean change (SMC) of pruritus measurements across the overall study population; the placebo response rate in the overall population; and the mean difference (MD) in Numerical Rating Scale (NRS) and Worst–Itch NRS (WI‐NRS) scores among patients with moderate‐to‐severe itching. We utilized the SMC to harmonize the disparate pruritus assessment scales employed across the included trials, while specific NRS and WI‐NRS analyses were conducted where sufficient raw data were available. When data availability permitted the analysis of the overall populations (SMC of pruritus measures and placebo response rate), we also performed a moderate‐to‐severe subgroup analysis. The overall randomized placebo population constituted the primary analysis for both continuous and dichotomous outcomes. Analyses restricted to participants with moderate‐to‐severe pruritus were considered subgroup analyses.

The placebo response rate was defined as the proportion of patients in the placebo arm who achieved a clinically meaningful reduction in pruritus intensity from baseline, based on validated or trial‐defined thresholds. For adult populations, this was consistently defined as a reduction of at least 3 points on a 0–10 NRS. In paediatric populations, response was defined as a reduction of at least 1 point on a 0–4 observer‐reported outcome scale, such as the ObsRO PRUCISION instrument.

Mean change from baseline was calculated as the endpoint value minus the baseline value; negative changes indicated improvement in pruritus. Across all studies, individuals with moderate‐to‐severe symptoms were defined as those with a baseline score of 4 or higher on the relevant pruritus assessment scale for adult patients. For paediatric trials, moderate‐to‐severe symptoms were defined according to trial‐specific thresholds of ≥ 1.5 or ≥ 2 on 0–4 observer‐ or patient‐reported PRUCISION/ItchRO scales. Further information regarding the detailed characteristics of inclusion criteria and methodological comparison of these pruritus assessment instruments is available in Table S3.

#### 2.4.2 Secondary Outcomes

We extracted additional quantitative pruritus metrics as secondary outcomes, including (1) the multidimensional 5‐D Itch Scale, which assesses duration, degree, direction, disability and distribution; and (2) the PBC‐40 overall score and itch domain among patients with PBC when reported.

### 2.5 Statistical Analysis

All statistical analyses were performed using R (version 4.5.2). To estimate the placebo response rate, we conducted proportional meta‐analyses by pooling responder proportions across the placebo arms of the included trials. Placebo response rates were synthesized using logit‐transformed proportions within random‐effects models, with restricted maximum likelihood (REML) estimation and the Hartung–Knapp adjustment. For each trial, the number of placebo responders and the total number of participants assigned to placebo were used to calculate pooled proportions and corresponding 95% confidence intervals (CIs).

For continuous outcomes, such as the mean change in pruritus intensity from baseline, we used two complementary approaches. First, changes measured using different instruments—including the 0–10 Numerical Rating Scale (NRS), 0–4 paediatric observer‐reported scales and the 0–70 Adult ItchRO—were pooled using the standardized mean change based on change scores (SMC). This approach expresses change in standard‐deviation units and avoids assuming linear equivalence between instruments with different scale ranges and measurement properties. For each placebo group, the uncorrected standardized change was calculated by dividing the reported mean baseline‐to‐endpoint change by the corresponding standard deviation of the change scores. A Hedges‐type small‐sample correction based on the placebo‐arm sample size was then applied. Effect sizes and sampling variances were calculated using the escalc() function of the metafor package, with measure = ‘SMCC’, correct = TRUE and vtype = ‘LS’. The vtype = ‘LS’ option specifies the large‐sample sampling‐variance estimator for the Hedges‐corrected standardized change.

Because the standard deviations of the change scores were extracted directly from the trial reports, no baseline‐to‐endpoint within‐person correlation was assumed or imputed. The paired nature of the baseline and endpoint measurements was already incorporated into the reported change‐score variability. Effect estimates were oriented so that negative values consistently indicated improvement in pruritus. The resulting effect estimates and standard errors were pooled using generic inverse‐variance random‐effects models implemented with the metagen() function of the meta package.

Second, where sufficient data were available, instrument‐specific changes were pooled in their original units using MDs. WI‐NRS and other NRS‐based measures were analysed separately because worst–itch and averaged itch assessments may differ systematically and were therefore not combined using raw mean differences. Both types of measures could contribute to the primary SMC analysis, whereas instrument‐specific MD analyses were conducted separately for WI‐NRS and other NRS‐based outcomes. Owing to data availability, these instrument‐specific analyses included only patients with a baseline NRS or WI‐NRS score of 4 or higher. In all continuous analyses, the standard deviation of the change from baseline was extracted directly from the reported trial data.

To account for clinical and methodological heterogeneity, all continuous meta‐analyses were performed using random‐effects models. Between‐study variance was estimated using REML, and confidence intervals for pooled estimates were calculated using the Hartung–Knapp adjustment. This adjustment was applied to account for uncertainty in the estimated between‐study variance, particularly given the limited number of studies contributing to some analyses.

Exploratory subgroup analyses were conducted according to clinically relevant study‐level characteristics when sufficient data were available. Age group, classified as adults versus children, was evaluated for both the continuous SMC outcome and the placebo response‐rate outcome. For the SMC outcome, additional subgroup analyses examined underlying liver disease, treatment duration using a 5‐month cut‐off to distinguish shorter‐ and longer‐duration trials, geographical scope and risk of bias. Corresponding subgroup analyses of the placebo response rate were not undertaken when responder definitions were incompatible or when the resulting subgroups contained too few studies for meaningful interpretation. Drug class was not evaluated because the mechanism of the active intervention was not considered an independent determinant of within‐placebo‐arm change. Geographical scope was classified as multinational or multicentre versus single‐country or single‐centre, where applicable. Country‐specific analyses were not conducted because most trials recruited participants across multiple countries.

For the secondary PBC‐40 and 5‐D Itch outcomes, mean changes from baseline and their corresponding measures of precision were extracted in the original instrument units. These analyses were restricted to populations with moderate‐to‐severe pruritus because only such data were available. When the PBC‐40 total‐score change or its corresponding standard deviation was not reported, as occurred in the ELATIVE trial [17], these values were derived from the reported changes in the six individual PBC‐40 domains. The mean change in the total score was calculated as the sum of the six domain‐level mean changes. Because the domains were measured in the same participants, their variances could not be treated as independent. The variance of the total change was therefore calculated by adding the six domain‐specific change‐score variances and twice the sum of the 15 pairwise covariance terms among the domains. As the correlations between domain‐level changes were not reported, each covariance was approximated using a common correlation coefficient of 0.5, as recommended by the Cochrane Handbook for Systematic Reviews of Interventions [18], multiplied by the product of the corresponding domain‐specific change‐score standard deviations. The standard deviation of the total change was obtained as the square root of the resulting total variance. This derivation was required only for the ELATIVE trial [17]; the other studies reported the PBC‐40 total‐score change and its corresponding variability directly.

Heterogeneity was assessed using the between‐study variance (*τ* ^2^), Cochran's *Q* test and the *I* ^2^ statistic. *I* ^2^ values below 50%, between 50% and 75%, and above 75% were considered to indicate low, moderate and high statistical heterogeneity, respectively. However, *I* ^2^ was interpreted conservatively, particularly in proportional meta‐analyses and analyses containing few studies, because elevated values may partly reflect the statistical properties of proportional data rather than substantive clinical or methodological inconsistency [19].

To identify potential clinical and methodological determinants of the placebo response, we performed exploratory univariable mixed‐effects meta‐regression analyses using the rma.uni() function of the metafor package (version 4.8.0), with REML estimation and Knapp–Hartung inference. The Hedges‐corrected SMC estimates and their corresponding sampling variances, calculated as described above, were used as the effect‐size data. The influence of each covariate was assessed using the omnibus test of moderators, which evaluated the null hypothesis that the moderator coefficient was zero and therefore whether the moderator was associated with variation in effect size. Residual heterogeneity following inclusion of the moderator was assessed using the residual between‐study variance (*τ* ^2^) and the residual *Q* statistic (QE). Analyses were restricted to moderators available in at least 10 studies, and the number of studies contributing to each model was reported explicitly. No multivariable meta‐regression models were fitted. Given the limited number of eligible trials and the use of aggregate study‐level data, all meta‐regression analyses were considered exploratory and hypothesis‐generating. Evaluated moderators included follow‐up duration, mean age, percentage of female participants, concurrent UDCA use, prior UDCA use and total bilirubin (TB). Baseline pruritus severity was also considered as a potential moderator. However, baseline scores were reported using instruments with substantially different ranges and measurement properties, including 0–10 NRS‐based measures, 0–4 paediatric observer‐reported scales and the 0–70 Adult ItchRO. Although changes measured using these instruments could contribute to the SMC outcome after standardization, the raw baseline scores remained expressed in their original instrument units and could not be interpreted as equivalent moderator values.

### 2.6 Risk of Bias Assessment

Risk of bias was assessed independently for each trial by two reviewers (L.C.S. and B.F.M.) using the Cochrane Risk of Bias tool (RoB 2) [20]. Risk‐of‐bias assessments were performed separately for the primary and secondary outcomes. Discrepancies were resolved through consensus or, when required, by consultation with a third reviewer (I.B.S.). Traffic‐light plots of the risk‐of‐bias assessments were generated.

### 2.7 Confidence in the Cumulative Evidence

We assessed the certainty of evidence for all outcomes, including pooled pruritus changes (SMC of pruritus measurements, WI‐NRS, NRS, 5‐D Itch and PBC‐40) and placebo response rate, using the Grading of Recommendations Assessment, Development, and Evaluation (GRADE) [21] framework. Within the PBC‐40 analyses, the itch‐domain and overall‐score outcomes were graded separately. Because these syntheses represented non‐comparative within‐placebo‐arm changes or proportions rather than randomized between‐group effects, certainty ratings were interpreted as confidence in the pooled descriptive estimates and not as evidence of a causal placebo effect. Heterogeneity was evaluated conservatively. For the placebo response rate, *I* ^2^ statistics in proportional meta‐analyses can overestimate true data inconsistency [19]. For continuous outcomes, elevated *I* ^2^ was not considered sufficient by itself to justify downgrading because these were descriptive within‐placebo‐arm syntheses analysed using random‐effects models; differences in populations, instruments and reporting sources were considered separately under indirectness where applicable. Therefore, no outcome was downgraded for inconsistency solely on the basis of heterogeneity statistics.

## 3 Results

### 3.1 Study Selection

Of the 3027 records initially identified, 904 were removed as duplicates. Screening the remaining titles and abstracts led to the exclusion of 1892 records. We performed a full‐text review of 231 reports, resulting in a final inclusion of 13 studies for the systematic review and meta‐analysis. Additionally, the supplementary search identified 991 records, from which 365 duplicates were removed. Of the remaining records, 626 were screened by title and abstract and 51 were assessed for full‐text eligibility, yielding no additional eligible trials. PRISMA flowcharts detailing the complete study selection processes for both searches are available in Figures S1 and S2.

### 3.2 Baseline Characteristics

Across the 13 included randomized controlled trials, 548 patients were assigned to placebo, comprising 394 patients with PBC, 66 with PFIC, 36 with mixed cholestatic diseases (PSC, PBC or SSC), 29 with Alagille syndrome and 23 with PSC. Overall, 459 participants were female (83.8%). At the trial level, most studies enrolled patients with PBC (*n* = 7), alongside two trials each for Alagille syndrome and PFIC, and one trial for PSC. GLISTEN trial [22] was the only trial with a subsequent crossover phase; only parallel‐group Part A data before any crossover were included.

Three trials used adult NRS measures, three used the WI‐NRS, one used the 0–70 Adult ItchRO, and one used a visual analogue scale. Four paediatric trials used 0–4 ItchRO/PRUCISION measures; two of these trials reported both observer‐ and patient‐reported versions, whereas two used observer‐reported measures only. One trial did not report a unidimensional pruritus‐intensity measure suitable for inclusion in the primary continuous analysis. Eight trials exclusively required at least moderate pruritus or scratching at enrolment, using instrument‐specific thresholds. The remaining trials enrolled broader populations or reported separate results for participants with moderate‐to‐severe pruritus. Nine trials evaluated adult populations, with mean ages ranging from 47.1 to 59.2 years, whereas four trials enrolled paediatric patients with Alagille syndrome or PFIC, with mean ages ranging from 2.8 to 5.5 years. Among studies reporting baseline fibrosis severity, 84 patients had advanced fibrosis or cirrhosis, corresponding to 15.3% of the total placebo cohort.

Baseline pruritus severity varied substantially across studies and could not be directly compared because different instruments and eligibility thresholds were used, with mean baseline scores ranging from 1.4 to 5.6 on the NRS, 2.8 to 2.9 on the ItchRO (Obs), and 7.3 on the Visual Analogue Scale (VAS), while the Adult ItchRO recorded a mean of 51.8. In paediatric trials, mean observer‐reported pruritus scores were consistently reported around 3.0. Cholestatic markers were notably elevated, with mean baseline alkaline phosphatase (ALP) levels ranging from 192.1 to 685.4 U/L (with one study reporting 1.6 × ULN). A high proportion of participants were receiving concurrent UDCA at baseline, reflecting standard‐of‐care practices, with 507 out of 548 patients (92.5%) across the included trials receiving the therapy and individual study proportions ranging from 73.9% to 100%. Follow‐up duration across the studies ranged from a minimum of 0.8 months to a maximum of 13 months. Study characteristics are summarized in Table 1, and detailed eligibility criteria are provided in Table S3.

**TABLE 1:** Baseline demographic and clinical characteristics of the placebo‐treated participants in the included randomized controlled trials.

Study	Geographical scope	Publication year	Placebo group (*n*)	Disease/medication	Age (mean)	Female (*n*)	White (*n*)	Baseline pruritus (mean ± SD)	Concurrent UDCA (*n*)	Treatment duration (months)	Follow‐up (months)	Alkaline phosphatase (mean ± SD)
ENHANCE	Multicentre	2023	87	PBC/Seladelpar	55.9	85	80	NRS 2.9 ± 2.5	85	4.1 (2.7)^a^	3	293.4 ± 106.2
Hirschfield et al.	Multicentre	2024	65	PBC/Seladelpar	57	60	56	NRS 3 ± 3	61	12	12	313.8 ± 117.7
GLISTEN	Multicentre	2025	119	PBC/Linerixibat	57	113	74	WI‐NRS 7.36 (1.454)	117	5.5	6	192.1 ± 133.5
ELATIVE	Multicentre	2024	54	PBC/Elafibranor	56.4	52	46	WI‐NRS 3.2 ± 2.9	51	12	13	323.1 ± 198.6
GLIMMER	Multicentre	2023	36	PBC/Linerixibat	54.4	34	26	NRS 5.6 ± 2.00^b^ Mild 11, Moderate 14, Severe 11	32	2.7	4	1.6 ± 1.24 × ULN
ELMWOOD	Multicentre	2026	23	PSC/Elafibranor	47.1	10	22	WI‐NRS 1.4 ± 2	17	2.7	3	415.8 ± 234.6
Mayo et al.	Multicentre	2019	24	PBC/Maralixibat	52	23	—	Adult ItchRO 51.8 ± 12.14	24	3	3.3	264.9 ± 152.27
EPICS‐1	USA	2022	10	PBC/Saroglitazar	59.2	10	9	—	10	4	4	294.9 ± 73.3
FITCH	Multicentre	2021	36	PSC, PBC or SSC/Bezafibrate	50	23	—	VAS score 7.3 ± 2.3	28	0.7	0.8	397 ± 360
Shneider et al.	USA	2018	12	Alagille syndrome/Maralixibat	5.5	6	9	ItchRO (Obs) 2.8 ± 0.53	9	3	3.3	685.4 ± 305.7
PEDFIC 1	Multicentre	2022	20	PFIC/Odevixibat	2.8	8	17	Pruritus score 3 ± 0.47	18	6	6	—
ASSERT	Multicentre	2024	16	Alagille syndrome/Odevixibat	4.2	11	13	Mean scratching score 3 ± 0.6	16	6	6	—
MARCH‐PFIC	Multicentre	2024	46	PFIC/Maralixibat	4.7	24	34	ItchRO (Obs) 2.9 ± 0.8	39	6	6.5	513 ± 255

Abbreviations: Adult ItchRO, adult itch‐reported outcome; ItchRO (Obs), observer‐reported itch outcome; *N*, number/sample size; NRS, Numerical Rating Scale; PBC, primary biliary cholangitis; PFIC, progressive familial intrahepatic cholestasis; PSC, primary sclerosing cholangitis; SD, standard deviation; SSC, secondary sclerosing cholangitis; UDCA, ursodeoxycholic acid; ULN, upper limit of normal; VAS, Visual Analogue Scale.

^a^ Mean (SD) duration of exposure. Exposure duration varied due to the premature study closure, resulting in a broad range of individual treatment periods.

^b^ GLIMMER baseline was defined at the end of the initial 4‐week single‐blind placebo period (week 4, visit 3); the value shown is the mean (SD) worst daily itch score in the placebo group.

### 3.3 Primary Outcomes

#### 3.3.1 Pooled SMC in the Overall Population

The meta‐analysis of 12 studies, including 404 placebo‐treated participants, demonstrated a pooled SMC of −0.49 (95% CI, −0.72 to −0.27), indicating a placebo‐associated improvement in pruritus intensity from baseline. Statistical heterogeneity was substantial (*I* ^2^ = 69.9%), and the pooled effect was statistically significant under the random‐effects model (*p* < 0.01) (Figure 1).

**FIGURE 1:**
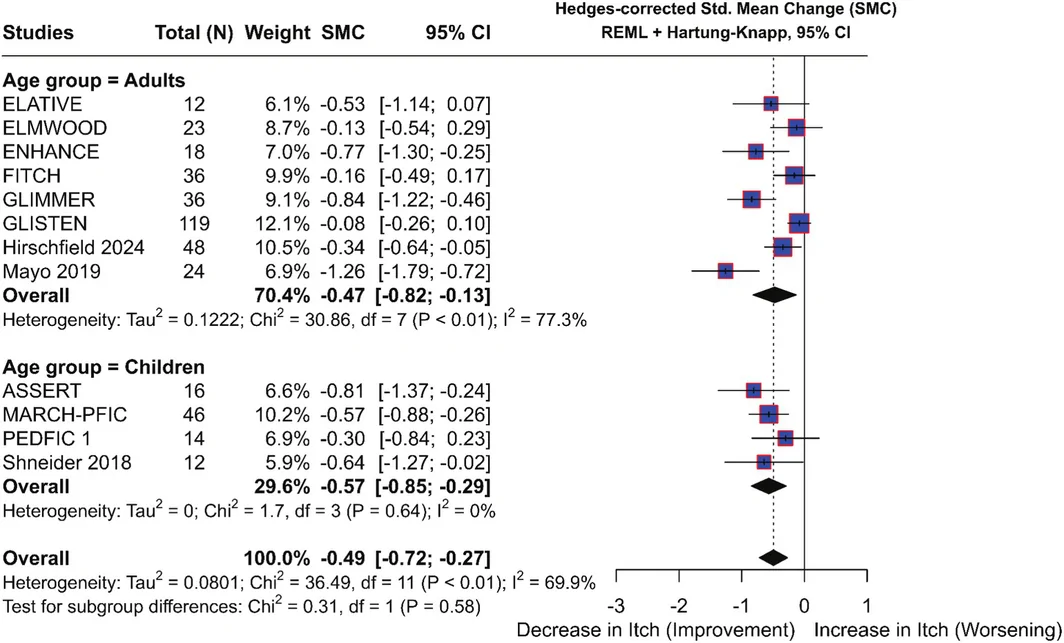
Forest plot of the standardized mean change based on change scores in pruritus intensity within placebo arms in the overall population, stratified by age group. Negative values indicate improvement in pruritus. These estimates do not represent comparisons between placebo and active treatment.

#### 3.3.2 Subgroup Analyses of the Pooled SMC in the Overall Population

There was no evidence that the placebo‐associated change differed according to age group, geographical scope, underlying liver disease, treatment duration or risk of bias, as all tests for subgroup differences were nonsignificant.

Stratification by age showed comparable estimates in adult trials (*k* = 8; SMC, −0.47; 95% CI, −0.82 to −0.13; *I* ^2^ = 77.3%) and paediatric trials (*k* = 4; SMC, −0.57; 95% CI, −0.85 to −0.29; *I* ^2^ = 0.0%). The test for subgroup differences was nonsignificant (*p* = 0.58) (Figure 1).

The placebo‐associated change was also similar across geographical settings. The single‐centre study conducted in the USA reported an SMC of −0.64 (95% CI, −1.27 to −0.02), whereas the 11 multicentre trials had a pooled SMC of −0.49 (95% CI, −0.73 to −0.24; *I* ^2^ = 72.0%). There was no evidence of a subgroup difference (*p* = 0.64) (Figure S3).

Subgroup analysis according to underlying liver disease did not identify a significant difference between disease categories (*p* = 0.08). Among the categories represented by multiple studies, the pooled SMC was −0.60 for PBC (*k* = 6; 95% CI, −1.04 to −0.16; *I* ^2^ = 82.7%) and −0.52 for PFIC (*k* = 3; 95% CI, −0.88 to −0.16; *I* ^2^ = 0.0%). Alagille syndrome, PSC, and mixed PBC, PSC or secondary sclerosing cholangitis were each represented by a single study and were also included in the test for subgroup differences (Figure S4).

Treatment duration was not associated with a significant difference in the placebo‐associated change (*p* = 0.27). Trials lasting < 5 months had a pooled SMC of −0.61 (*k* = 6; 95% CI, −1.07 to −0.15; *I* ^2^ = 73.9%), compared with −0.38 in trials lasting 5 months or longer (*k* = 6; 95% CI, −0.65 to −0.11; *I* ^2^ = 59.0%) (Figure S5).

Similarly, there was no evidence of a subgroup difference according to risk of bias (*p* = 0.68). Studies judged to be at low risk of bias had a pooled SMC of −0.53 (*k* = 10; 95% CI, −0.77 to −0.28; *I* ^2^ = 57.5%), whereas studies with some concerns had a pooled SMC of −0.38 (*k* = 2; 95% CI, −4.75 to 3.98; *I* ^2^ = 83.2%). The estimate for studies with some concerns was highly imprecise because it was based on only two heterogeneous studies (Figure S6).

#### 3.3.3 Pooled SMC in Participants With Moderate‐to‐Severe Pruritus

The analysis restricted to participants with moderate‐to‐severe pruritus at baseline included 11 studies and 337 placebo‐treated participants. The pooled SMC was −0.57 (95% CI, −0.81 to −0.33), indicating a significant placebo‐associated improvement from baseline. Statistical heterogeneity was substantial (*I* ^2^ = 72.0%; *p* < 0.01) (Figure 2).

**FIGURE 2:**
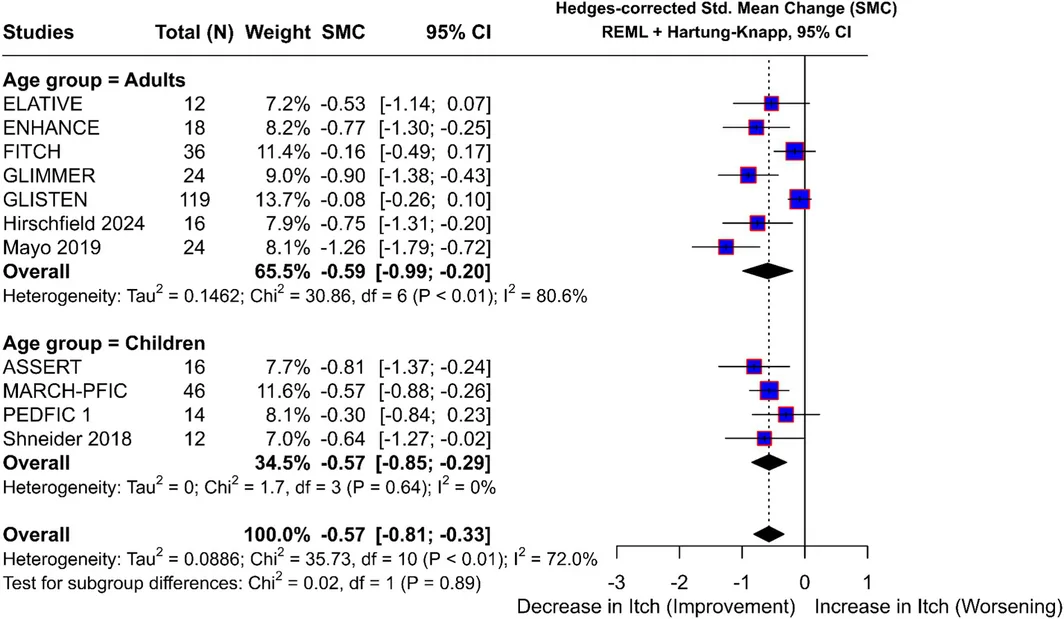
Forest plot of the standardized mean change based on change scores in pruritus intensity within placebo arms among participants with moderate‐to‐severe pruritus, stratified by age group. Negative values indicate improvement in pruritus. These estimates do not represent comparisons between placebo and active treatment.

#### 3.3.4 Subgroup Analyses in Participants With Moderate‐to‐Severe Pruritus

There was no evidence that the placebo‐associated change in the moderate‐to‐severe population differed according to age group, geographical scope, underlying liver disease, treatment duration or risk of bias.

The pooled SMC was −0.59 in adult trials (*k* = 7; 95% CI, −0.99 to −0.20; *I* ^2^ = 80.6%) and −0.57 in paediatric trials (*k* = 4; 95% CI, −0.85 to −0.29; *I* ^2^ = 0.0%). The test for subgroup differences was nonsignificant (*p* = 0.89) (Figure 2).

Geographical scope was also not associated with a significant subgroup difference (*p* = 0.83). The single‐centre study conducted in the USA reported an SMC of −0.64 (95% CI, −1.27 to −0.02), whereas the 10 multicentre trials had a pooled SMC of −0.57 (95% CI, −0.84 to −0.30; *I* ^2^ = 74.3%) (Figure S7).

Subgroup analysis according to underlying liver disease showed no significant difference between disease categories (*p* = 0.09). Among categories represented by multiple studies, the pooled SMC was −0.68 for PBC (*k* = 6; 95% CI, −1.12 to −0.24; *I* ^2^ = 83.1%) and −0.52 for PFIC (*k* = 3; 95% CI, −0.88 to −0.16; *I* ^2^ = 0.0%). Alagille syndrome and mixed PBC, PSC or secondary sclerosing cholangitis were each represented by a single study and were also included in the test for subgroup differences (Figure S8).

Treatment duration was not associated with a significant subgroup difference (*p* = 0.23). Trials lasting < 5 months had a pooled SMC of −0.72 (*k* = 5; 95% CI, −1.24 to −0.20; *I* ^2^ = 72.6%), whereas trials lasting 5 months or longer had a pooled SMC of −0.45 (*k* = 6; 95% CI, −0.76 to −0.14; *I* ^2^ = 66.1%) (Figure S9).

Finally, there was no evidence of a subgroup difference according to risk of bias (*p* = 0.49). Studies judged to be at low risk of bias had a pooled SMC of −0.63 (*k* = 9; 95% CI, −0.89 to −0.37; *I* ^2^ = 50.8%), whereas studies with some concerns had a pooled SMC of −0.38 (*k* = 2; 95% CI, −4.75 to 3.98; *I* ^2^ = 83.2%). The latter estimate was highly imprecise because only two heterogeneous studies contributed to this subgroup (Figure S10).

#### 3.3.5 Pooled Mean Change in Specific Pruritus Scales

In the analysis restricted to patients with moderate‐to‐severe pruritus at baseline, we assessed the placebo‐arm response using the WI‐NRS and the NRS. The meta‐analysis of three studies reporting on the WI‐NRS (*n* = 154) demonstrated a pooled mean change of −0.65 points (95% CI, −1.36 to 0.07; *I* ^2^ = 0.0%) (Figure S11). Similarly, the meta‐analysis of three studies reporting on the NRS (*n* = 58) showed a pooled mean reduction of −1.62 points (95% CI, −2.12 to −1.12; *I* ^2^ = 0.0%) (Figure S12).

#### 3.3.6 Placebo Response Rate

The primary categorical analysis evaluated the proportion of patients achieving a clinically meaningful placebo response across both the unselected total cohort and the moderate‐to‐severe symptom subpopulation. For the overall patient population (*k* = 9; *n* = 338), the pooled placebo response rate was 30.0% (95% CI, 20.6% to 41.4%; *I* ^2^ = 52.2%) (Figure 3). When the analysis was restricted to the moderate‐to‐severe subpopulation of patients across seven trials (*k* = 7; *n* = 227), the pooled placebo response rate in this baseline‐restricted subpopulation was 27.4% (95% CI, 17.9% to 39.5%; *I* ^2^ = 48.9%) (Figure S13).

**FIGURE 3:**
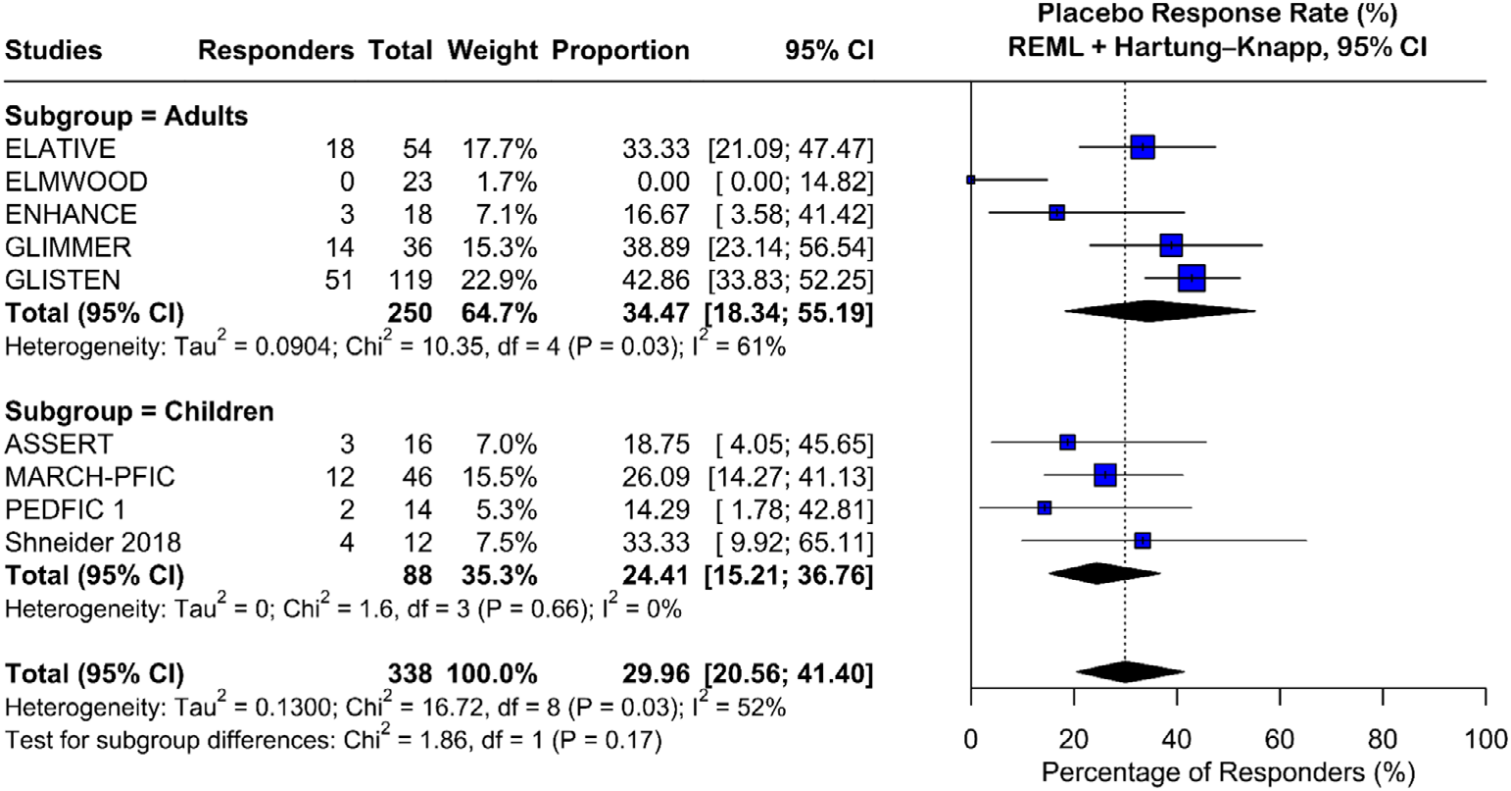
Forest plot of the placebo response rate in the overall placebo population, stratified by age group. Responder definitions were trial‐ and instrument‐specific and are detailed in Table S3. Estimates are pooled proportions within placebo arms.

#### 3.3.7 Subgroup Analyses of the Placebo Response Rate

In the overall population of nine trials, adult trials (*k* = 5) demonstrated a pooled placebo response rate of 34.4% (95% CI, 18.3% to 55.1%), whereas paediatric trials (*k* = 4) showed a response rate of 24.4% (95% CI, 15.2% to 36.7%), resulting in a *p*‐value for subgroup differences of 0.17 (Figure 3). Similarly, within the moderate‐to‐severe baseline subpopulation of seven trials, the pooled responder rate was 29.5% (95% CI, 5.3% to 75.5%) for adult studies (*k* = 3) compared to 24.4% (95% CI, 15.2% to 36.7%) for paediatric studies (*k* = 4), with *p* = 0.60 (Figure S13).

### 3.4 Secondary Outcomes

#### 3.4.1 Mean Change in 5‐D Itch Scale

The pooled MD from baseline in the multidimensional 5‐D Itch scale (*k* = 5; *n* = 110) was −1.72 (95% CI, −5.54 to 2.11), which did not reach statistical significance. Heterogeneity for this outcome was *I* ^2^ = 92.6% (*τ* ^2^ = 8.75, *Q* = 53.95, *p* < 0.0001) (Figure S14).

#### 3.4.2 Mean Change in PBC‐40 Itch Domain

Placebo arms contributing PBC‐40 itch‐domain data (*k* = 5; *n* = 172 observations) showed a statistically significant pooled reduction in the PBC‐40 itch domain, with a mean change of −2.02 points (95% CI, −3.76 to −0.28). Between‐study heterogeneity for this domain was *I* ^2^ = 83.4% (*τ* ^2^ = 1.61; *Q* = 24.05; *p* < 0.0001) (Figure 15A).

#### 3.4.3 Mean Change in PBC‐40 Overall Scale

The analysis of the PBC‐40 overall score (*k* = 5; *n* = 172 observations) also demonstrated a statistically significant placebo‐associated mean reduction. The pooled mean reduction was −9.13 points (95% CI, −15.28 to −2.98). Heterogeneity was *I* ^2^ = 78.1% (*τ* ^2^ = 19.13; *Q* = 18.27; *p* = 0.001) (Figure 15B).

### 3.5 Exploratory Meta‐Regression

Meta‐regression analyses were restricted to moderators available in at least 10 trials. Six study‐level moderators met the eligibility criterion: follow‐up duration, mean age, percentage of female participants, concurrent UDCA use, prior UDCA use and total bilirubin.

None of the evaluated moderators was significantly associated with the magnitude of the placebo‐associated change in pruritus intensity (Table S4). Residual heterogeneity remained statistically significant in all fitted models. Several other pre‐specified clinical and biochemical variables—including percentage of white participants, disease duration, history of pruritus and baseline NRS pruritus scores, and markers such as ALP, ALT, AST and GGT—were considered but could not be formally evaluated because they were reported in fewer than 10 trials. Given the limited number of studies and the aggregate‐data design, these analyses should be interpreted as exploratory and hypothesis‐generating.

### 3.6 Risk of Bias

Methodological quality was generally high across the included trials. For the primary outcome of SMC of pruritus measurements, 10 studies were assessed as having a low risk of bias, while two were identified as having some concerns (Figure S16). In the analysis of the placebo response rate, seven trials were categorized as low risk and two raised some concerns (Figure S17). For the WI‐NRS assessment, two studies were at low risk of bias, while one study raised some concerns (Figure S18). Similarly, for the NRS assessment, two studies were at low risk of bias, while one study raised some concerns (Figure S19). Regarding secondary outcomes, three trials evaluating the PBC‐40 (overall and itch domains) were at low risk of bias, with two raising some concerns (Figure S20). Finally, for the 5‐D Itch Scale, four studies were considered low risk and one was assessed as having some concerns (Figure S21).

### 3.7 Certainty of Evidence

The certainty of evidence was rated as moderate for the pooled SMC and for the PBC‐40 itch‐domain and overall‐score outcomes, and as low for the WI‐NRS, NRS, placebo response rate, and 5‐D Itch outcomes. No outcome was downgraded for inconsistency solely on the basis of elevated heterogeneity statistics. The SMC was downgraded for serious indirectness because it combined different cholestatic diseases, adult and paediatric populations, instruments with different constructs and ranges, and both patient‐ and observer‐reported measures. The placebo response rate was downgraded for serious indirectness because adult and paediatric trials used non‐equivalent response definitions and reporting sources, and for serious imprecision because its 95% CI ranged from 20.6% to 41.4%. The WI‐NRS, NRS and 5‐D Itch outcomes were rated as low because of very serious imprecision, whereas the PBC‐40 itch‐domain and overall‐score outcomes were rated as moderate after a single downgrade for serious imprecision. These ratings apply to pooled descriptive within‐placebo‐arm estimates and do not establish a causal placebo effect distinct from natural history, regression to the mean or other effects of trial participation. The complete GRADE assessment and outcome‐specific rationales are provided in Table S5.

## 4 Discussion

### 4.1 Overview

In this systematic review and meta‐analysis, we found that the placebo response is substantial in randomized trials of contemporary therapies for cholestatic pruritus. Across the included trials, the pooled SMC for pruritus reduction was −0.49 (95% CI, −0.72 to −0.27), and approximately 30% of the overall placebo population achieved a clinically meaningful improvement in pruritus intensity. In practical terms, nearly one in three placebo‐treated patients experienced a meaningful reduction in itch intensity despite receiving no active therapy. These findings underscore the placebo response as a central determinant of endpoint performance and the interpretation of therapeutic efficacy. Furthermore, we observed that the magnitude of this response is highly sensitive to the specific assessment instrument employed: while some unidimensional scales like the NRS showed significant placebo‐associated reductions—with pooled mean decreases of −1.62 points (*k* = 3)—other instruments, including the WI‐NRS (*k* = 3) and the multidimensional 5‐D Itch Scale (*k* = 5), failed to demonstrate statistical significance. Disease‐specific quality‐of‐life instruments such as the PBC‐40 (which showed a significant pooled reduction of −9.13 points in the overall score and −2.02 in the itch domain across 5 studies) appear more susceptible to the contextual effects of the placebo response than the 5‐D Itch Scale, highlighting a notable divergence in how different instruments capture symptom improvement. Notably, exploratory meta‐regression did not identify any statistically significant study‐level moderator of the standardized placebo‐associated change.

### 4.2 Comparison With the Literature

#### 4.2.1 Comparison With Dermatological Conditions

The 27.4% responder rate identified in our baseline‐restricted subpopulation and approximately 30% in the overall population is substantially higher than the 14% and 5% rates (PASI‐50 and PASI‐75, respectively) typically reported in psoriasis trials [23]. This discrepancy likely reflects the inherent subjectivity of pruritus compared to the objective clinical assessment of skin lesions. While our results are more closely aligned with atopic dermatitis, where placebo arms show a 14.1% mean reduction in the NRS itch scale [24], our observed placebo response rate remains notably higher. Furthermore, a key distinction emerges in the moderators of these responses: while atopic dermatitis trials found that higher baseline severity and male sex increased the placebo effect [24], our study found no demographic moderation. In the present analysis, we did not detect significant associations with mean age or the percentage of female participants. Similarly, follow‐up duration, concurrent UDCA use, prior UDCA use and baseline total bilirubin were not statistically significant moderators.

#### 4.2.2 Assessment Tool Divergence

Placebo‐associated change appeared to vary according to the outcome instrument used. The PBC‐40 overall score and its itch domain showed statistically significant placebo‐associated changes from baseline, with pooled mean reductions of −9.13 points and −2.02 points, respectively. Notably, this is a validated, disease‐specific measure explicitly developed to capture the unique symptom burden of primary biliary cholangitis [25]. Conversely, the 5‐D Itch Scale, a multidimensional pruritus instrument, did not show a statistically significant change from baseline (pooled mean change of −1.72 points).

This variation across secondary metrics holds important methodological implications. While broad health‐related quality‐of‐life (HRQOL) measures like the PBC‐40 are susceptible to the psychosocial impact of trial participation (the Hawthorne effect) [25], the 5‐D Itch Scale is designed to offer specific resistance to placebo‐driven fluctuations by capturing broader and potentially more stable dimensions of chronic pruritus, including degree, duration, direction, disability and distribution [26]. The lack of statistical significance observed only for the 5‐D Itch Scale suggests that this multidimensional structure may inherit a higher threshold for non‐specific clinical changes than either single‐item intensity metrics or quality‐of‐life instruments. However, it is important to highlight that the non‐significant findings for the 5‐D Itch Scale may also reflect limited statistical power due to fewer reporting studies (*k* = 5) and variations in baseline symptom domains. Accordingly, these findings should be viewed as exploratory, supporting the need for future trials to prospectively evaluate how different patient‐reported outcome instruments capture placebo response in cholestatic pruritus.

#### 4.2.3 Natural Symptom Variability and Biochemical Severity

None of the six study‐level variables evaluated in the exploratory meta‐regression significantly moderated the placebo‐associated change in pruritus intensity, and residual heterogeneity remained statistically significant across all models. This finding suggests that variations in placebo responses may be driven by uncaptured factors, such as symptom variability, patient expectations, trial–site interactions, rescue medication policies, adherence, run‐in periods, recall windows, assessment frequency, regression to the mean or instrument psychometric differences.

Previous studies have shown that cholestatic pruritus can fluctuate substantially in both severity and duration over the course of disease, and that its apparent trajectory may vary according to the outcome measure used. In a 48‐week prospective cohort of patients with PSC, Hussain et al. [27] reported that, among those with moderate‐to‐severe pruritus, 61.5% experienced persistently intense symptoms, whereas 46.2% and 41.4% had spontaneous reductions of at least 2 points in worst–itch NRS and average‐itch NRS scores, respectively, without initiation of a new antipruritic therapy. These findings are directionally consistent with the substantial improvement observed in the placebo arms of our meta‐analysis, including a pooled response rate of approximately 30% and a significant mean reduction of 1.62 points on the NRS.

Gungabissoon et al. [28] provide further context for our WI‐NRS findings. In their 6‐month longitudinal cohort, most participants with moderate‐to‐severe pruritus at baseline remained within the same severity category across chronic liver diseases, and WI‐NRS scores did not change significantly over time. This pattern is consistent with our meta‐analysis, in which the pooled placebo‐associated reduction in WI‐NRS was −0.65 points and did not reach statistical significance. Kowdley et al. [29] similarly highlighted the fluctuating nature of cholestatic pruritus, with patients reporting episodes lasting from days to years and varying considerably in intensity, which may partly account for the substantial placebo‐associated improvement observed in clinical trials.

Collectively, these findings suggest that natural symptom variability, endpoint selection, assessment timing and underlying disease severity should all be considered when interpreting improvement within placebo arms and when defining expected control‐group response assumptions for future trials. Furthermore, the absence of significant trial‐level associations may reflect genuinely limited moderation, or it could stem from a small number of eligible trials, restricted between‐study variation, covariate imprecision and residual confounding.

### 4.3 Clinical and Research Implications

Our findings have important implications for the design and interpretation of clinical trials in cholestatic pruritus. The observation that placebo‐associated changes were statistically significant for unidimensional scales like the NRS (*k* = 3) and disease‐specific instruments like the PBC‐40 (*k* = 5), but failed to reach significance for the multidimensional 5‐D Itch Scale (*k* = 5), highlights the importance of endpoint architecture. Single‐item intensity scales and disease‐specific domains may be highly responsive to changes in symptom perception and expectation. In contrast, certain multidimensional instruments may capture more stable constructs, including functional impairment and chronicity. Therefore, these instruments should not be viewed as interchangeable measures of the same phenomenon, but as complementary tools capturing different layers of the pruritus experience.

The meta‐regression findings do not currently support the use of mean age, sex distribution, follow‐up duration, UDCA exposure or baseline total bilirubin as established stratification variables for controlling placebo response. Hussain et al. [27] associated greater pruritus intensity with cirrhosis, liver stiffness and biochemical severity. This apparent difference suggests that clinically relevant individual‐level associations may be obscured in analyses based on aggregate study‐level data. Trialists should continue to collect and report these variables consistently so that their potential roles can be assessed in larger datasets and individual participant‐level analyses.

Given the stability of the placebo effect across different follow‐up durations, trialists might consider moving beyond simple parallel‐group designs. Recent large‐scale Phase 3 structures [22] suggest that incorporating a blinded crossover or randomized withdrawal phase (e.g., switching patients from active treatment to placebo after an initial period) can provide valuable evidence. Observing whether symptoms worsen upon switching to placebo—or improve upon switching to the active drug—can help validate the true therapeutic gain of an intervention.

Finally, these pooled placebo estimates may also serve as empirically derived assumptions for sample‐size calculations in future cholestatic pruritus trials. Underestimating placebo response may result in underpowered studies, whereas incorporating realistic placebo response rates may improve trial design, statistical power and interpretation of treatment efficacy.

Future trials may also benefit from incorporating objective or semi‐objective digital tools to complement patient‐reported pruritus outcomes. Wearable sensors, actigraphy, accelerometers or other devices capable of capturing scratching behaviour, nocturnal activity and sleep disruption may provide complementary measures of itch burden that are less dependent on subjective recall or expectation.

### 4.4 Limitations

This meta‐analysis has limitations that warrant consideration. First, the number of eligible randomized controlled trials was modest (*k* = 13), which limited our statistical power to detect all potential clinical or methodological moderators of the placebo response. This constraint was particularly acute for the primary and secondary outcomes—such as the NRS (*k* = 3), WI‐NRS (*k* = 3), 5‐D Itch Scale (*k* = 5) and PBC‐40 overall score (*k* = 5)—where the small number of studies likely contributed to the wide confidence intervals observed, limiting the ability to draw definitive conclusions regarding the magnitude of placebo‐associated changes. However, this should be interpreted in the context of cholestatic liver diseases, which are rare disorders with a limited number of completed randomized trials.

Second, the analysed populations varied across different components of the study. While our primary outcomes—the pooled SMC of pruritus measurements and the placebo response rate—were assessed across the overall and moderate‐to‐severe populations, our specific MD analyses for NRS and WI‐NRS, as well as the secondary PBC‐40 and 5‐D Itch outcomes, were restricted only to patients with moderate‐to‐severe pruritus (baseline NRS or WI‐NRS ≥ 4) to address data availability. Standardization addresses differences in scale units but does not eliminate heterogeneity related to construct definition, instrument reliability, reporting method or population variability. Pooled estimates across patient‐reported and observer‐reported instruments should therefore be interpreted cautiously. Because the SMC is expressed in standard‐deviation units, its clinical interpretation is less direct than that of an instrument‐specific mean difference. Differences in study‐level standard deviations may also reflect true differences in population variability rather than only differences between measurement scales. Additionally, our exploratory meta‐regression analyses were conducted using data from the overall placebo cohorts of each trial. These variations in population focus were pragmatic decisions driven by the limited availability of granular subgroup data in published trial reports.

Third, we observed significant heterogeneity in the pruritus assessment instruments used across trials, including the NRS, WI‐NRS, VAS, ItchRO and PRUCISION scales (Table S3). Direct comparisons across these different pruritus assessment instruments are limited by differences in scale range (e.g., 0–10 vs. 0–4) and construct properties (e.g., peak intensity versus observer‐reported scratching behaviour). Consequently, these variations necessitate a conservative interpretation of pooled effect estimates across disparate assessment tools.

Fourth, the meta‐regression findings should be considered hypothesis‐generating. To reduce the risk of overfitting and unstable estimates, we restricted meta‐regression to covariates reported in at least 10 studies. Consequently, several pre‐specified clinical and biochemical variables—including the percentage of white participants, disease duration, history of pruritus, baseline NRS scores and markers such as ALP, ALT, AST and GGT—were excluded from meta‐regression due to insufficient reporting across the included trials.

Finally, because this study relied on aggregate published data, we could not assess individual‐level determinants of placebo response, including symptom variability, prior treatment exposure, rescue medication use, adherence, sleep disturbance, psychological factors or patient expectations. Future individual participant‐level analyses using standardized pruritus endpoints and harmonized trial reporting are needed to validate these findings and improve placebo assumptions for future sample‐size calculations.

## 5 Conclusions

Placebo‐associated improvement was substantial but differed across pruritus outcome instruments. These differences may influence assay sensitivity and the estimated net benefit of active therapy. The NRS demonstrated a statistically significant reduction, whereas the WI‐NRS and 5‐D Itch estimates did not reach statistical significance. PBC‐40 overall and itch domain scores showed significant placebo‐associated reductions.

These findings indicate that placebo response is not merely background noise, but a central determinant of trial interpretation, endpoint performance and perceived therapeutic benefit. The estimates provided here may inform sample‐size calculations, instrument selection and future trial designs aimed at distinguishing pharmacological efficacy from nonspecific improvement in cholestatic pruritus.

## Author Contributions

**Gustavo André Pedral Diniz Leite:** data curation, investigation, writing – original draft, writing – review and editing. **Pedro Robson Costa Passos:** formal analysis, writing – original draft, writing – review and editing. **Igor Boechat Silveira:** conceptualization, data curation, formal analysis, investigation, methodology, project administration, writing – original draft, writing – review and editing. **Sarah Quick Lourenço de Lima:** investigation, writing – original draft, writing – review and editing. **Gabriel André Pedral Diniz Leite:** writing – original draft, writing – review and editing, investigation. **Bernardo de Faria Moraes:** investigation, writing – original draft, writing – review and editing. **Guilherme Grossi Lopes Cançado:** conceptualization, formal analysis, methodology, writing – review and editing, supervision, writing – original draft. **Milena Ramos Tomé:** data curation, investigation, writing – original draft, writing – review and editing. **Leonardo Corrêa Süffert:** conceptualization, investigation, writing – original draft, writing – review and editing.

## Funding

The authors have nothing to report.

## Conflicts of Interest

The authors declare no conflicts of interest.

## Supporting information


**Figure S1:** Preferred Reporting Items for Systematic Reviews and Meta‐Analyses flowchart detailing the primary study selection process from initial identification to final inclusion.

**Figure S2:** Preferred Reporting Items for Systematic Reviews and Meta‐Analyses flowchart detailing the additional study selection process from initial identification to final inclusion.

**Figure S3:** Subgroup analysis of the standardized mean change in pruritus intensity stratified by geographical scope. Negative values indicate improvement in pruritus. These estimates do not represent comparisons between placebo and active treatment.

**Figure S4:** Subgroup analysis of the standardized mean change in pruritus intensity stratified by underlying disease. Negative values indicate improvement in pruritus. These estimates do not represent comparisons between placebo and active treatment.

**Figure S5:** Subgroup analysis of the standardized mean change in pruritus intensity stratified by treatment duration (< 5 months vs. ≥ 5 months). Negative values indicate improvement in pruritus. These estimates do not represent comparisons between placebo and active treatment.

**Figure S6:** Subgroup analysis of the standardized mean change in pruritus intensity stratified by risk of bias. Negative values indicate improvement in pruritus. These estimates do not represent comparisons between placebo and active treatment.

**Figure S7:** Subgroup analysis of the standardized mean change in moderate‐to‐severe pruritus stratified by geographical scope. Negative values indicate improvement in pruritus. These estimates do not represent comparisons between placebo and active treatment.

**Figure S8:** Subgroup analysis of the standardized mean change in moderate‐to‐severe pruritus stratified by underlying disease. Negative values indicate improvement in pruritus. These estimates do not represent comparisons between placebo and active treatment.

**Figure S9:** Subgroup analysis of the standardized mean change in moderate‐to‐severe pruritus stratified by treatment duration. Negative values indicate improvement in pruritus. These estimates do not represent comparisons between placebo and active treatment.

**Figure S10:** Subgroup analysis of the standardized mean change in moderate‐to‐severe pruritus stratified by risk of bias. Negative values indicate improvement in pruritus. These estimates do not represent comparisons between placebo and active treatment.

**Figure S11:** Forest plot of the pooled mean change from baseline in the Worst–Itch NRS (WI‐NRS) for patients with moderate‐to‐severe pruritus. Negative values indicate improvement in pruritus. These estimates do not represent comparisons between placebo and active treatment.

**Figure S12:** Forest plot of the pooled mean change from baseline in the Numerical Rating Scale (NRS) for patients with moderate‐to‐severe pruritus. Negative values indicate improvement in pruritus. These estimates do not represent comparisons between placebo and active treatment.

**Figure S13:** Subgroup analysis of the placebo response rate in the moderate‐to‐severe subpopulation stratified by age group. Responder definitions were trial‐ and instrument‐specific and are detailed in Table S3. Estimates are pooled proportions within placebo arms.

**Figure S14:** Forest plot of the pooled mean difference from baseline in the 5‐D Itch scale. Negative values indicate improvement in pruritus. These estimates do not represent comparisons between placebo and active treatment.

**Figure S15:** (A) Forest plot illustrating the pooled mean change in the PBC‐40 itch domain. Negative values indicate improvement in pruritus. These estimates do not represent comparisons between placebo and active treatment. (B) Forest plot illustrating the pooled mean change in the PBC‐40 overall score. Negative values indicate improvement in pruritus. These estimates do not represent comparisons between placebo and active treatment.

**Figure S16:** Risk of bias assessment for the standardized mean change of pruritus intensity.

**Figure S17:** Risk of bias assessment for the placebo response rate.

**Figure S18:** Risk of bias assessment for the mean difference in Worst–Itch NRS (WI‐NRS).

**Figure S19:** Risk of bias assessment for the mean difference in Numerical Rating Scale (NRS).

**Figure S20:** Risk of bias assessment for the secondary outcomes of the PBC‐40 overall score and itch domain.

**Figure S21:** Risk of bias assessment for the 5‐D Itch score.

**Table S1:** Preferred Reporting Items for Systematic Reviews and Meta‐Analyses 2020 checklist.

**Table S2:** Detailed search strategy and search results for the databases.

**Table S3:** Clinical eligibility criteria and pruritus assessment methodology for included randomized controlled trials.

**Table S4:** Summary of exploratory mixed‐effects meta‐regression models evaluated to identify potential determinants of the placebo response.

**Table S5:** Assessment of the certainty of evidence for each analysed outcome using the Grading of Recommendations Assessment, Development and Evaluation framework.

## Data Availability

The data that support the findings of this study are available from the corresponding author upon reasonable request.
