# Supplementary material for: Meta‐Analysis: The Placebo Response in Modern Treatments for Cholestatic Pruritus: A Systematic Review and Meta‐Analysis of Randomized Controlled Trials
Source: Aliment Pharmacol Ther. 2026 Sep 24;64(10):1348–61. doi: 10.1111/apt.70915 (PMC13688708; doi:10.1111/apt.70915)

Studies	Placebo Response Mean Change	SD	Total	Weight	Pooled Estimate	95% CI
ENHANCE	-1.55	1.91	18	31.4%	-1.55	[-2.43; -0.67]
GLIMMER	-2.10	2.25	24	30.4%	-2.10	[-2.99; -1.20]
Hirschfield 2024	-1.30	1.64	16	38.2%	-1.30	[-2.10; -0.50]
**Total (95% CI)**			**58**	**100.0%**	**-1.62**	**[-2.12; -1.12]**

Heterogeneity: $Tau^2 = 0$; $Chi^2 = 1.72$, df = 2 (P = 0.42); $I^2 = 0\%$

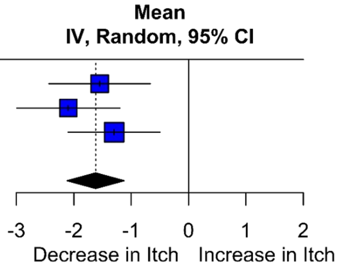

Supplement: Supplementary file 1 [file APT-64-1348-s002.docx]
