# Supplementary material for: Meta‐Analysis: The Placebo Response in Modern Treatments for Cholestatic Pruritus: A Systematic Review and Meta‐Analysis of Randomized Controlled Trials
Source: Aliment Pharmacol Ther. 2026 Sep 24;64(10):1348–61. doi: 10.1111/apt.70915 (PMC13688708; doi:10.1111/apt.70915)

Studies	Total (N)	Weight	SMC	95% CI
**Underlying liver disease = Alagille**				
ASSERT	16	7.7%	-0.81	[-1.37; -0.24]
**Underlying liver disease = PBC**				
ELATIVE	12	7.2%	-0.53	[-1.14; 0.07]
ENHANCE	18	8.2%	-0.77	[-1.30; -0.25]
GLIMMER	24	9.0%	-0.90	[-1.38; -0.43]
GLISTEN	119	13.7%	-0.08	[-0.26; 0.10]
Hirschfield 2024	16	7.9%	-0.75	[-1.31; -0.20]
Mayo 2019	24	8.1%	-1.26	[-1.79; -0.72]
**Overall**		**54.1%**	**-0.68**	**[-1.12; -0.24]**
Heterogeneity: $Tau^2 = 0.1436$; $Chi^2 = 29.64$, df = 5 ($P < 0.01$); $I^2 = 83.1\%$				
**Underlying liver disease = PBC, PSC, or SSC**				
FITCH	36	11.4%	-0.16	[-0.49; 0.17]
**Underlying liver disease = PFIC**				
MARCH-PFIC	46	11.6%	-0.57	[-0.88; -0.26]
PEDFIC 1	14	8.1%	-0.30	[-0.84; 0.23]
Shneider 2018	12	7.0%	-0.64	[-1.27; -0.02]
**Overall**		**26.8%**	**-0.52**	**[-0.88; -0.16]**
Heterogeneity: $Tau^2 = 0$; $Chi^2 = 0.88$, df = 2 ($P = 0.64$); $I^2 = 0\%$				
**Overall**		**100.0%**	**-0.57**	**[-0.81; -0.33]**
Heterogeneity: $Tau^2 = 0.0886$; $Chi^2 = 35.73$, df = 10 ($P < 0.01$); $I^2 = 72.0\%$				
Test for subgroup differences: $Chi^2 = 6.37$, df = 3 ($P = 0.09$)				

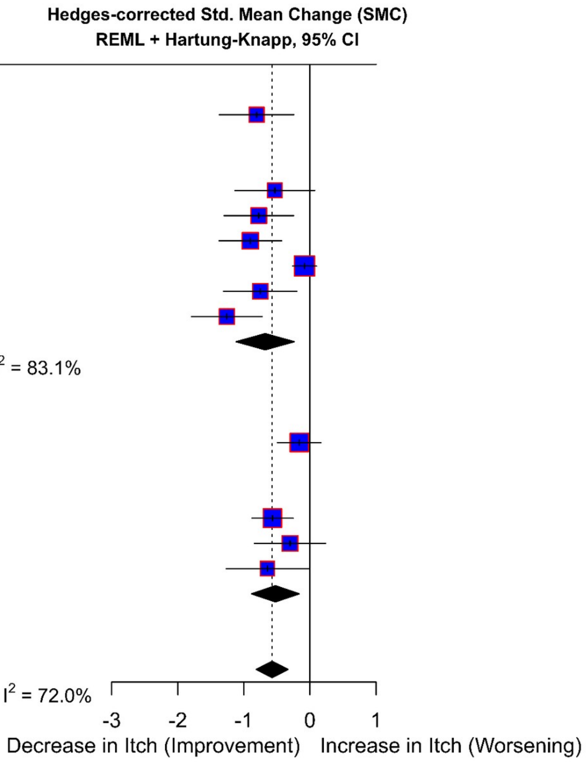

Supplement: Supplementary file 2 [file APT-64-1348-s004.docx]
