# Supplementary material for: Meta‐Analysis: The Placebo Response in Modern Treatments for Cholestatic Pruritus: A Systematic Review and Meta‐Analysis of Randomized Controlled Trials
Source: Aliment Pharmacol Ther. 2026 Sep 24;64(10):1348–61. doi: 10.1111/apt.70915 (PMC13688708; doi:10.1111/apt.70915)

Studies	Total (N)	Weight	SMC	95% CI
**Underlying liver disease = Alagille**				
ASSERT	16	6.6%	-0.81	[-1.37; -0.24]
**Underlying liver disease = PBC**				
ELATIVE	12	6.1%	-0.53	[-1.14; 0.07]
ENHANCE	18	7.0%	-0.77	[-1.30; -0.25]
GLIMMER	36	9.1%	-0.84	[-1.22; -0.46]
GLISTEN	119	12.1%	-0.08	[-0.26; 0.10]
Hirschfield 2024	48	10.5%	-0.34	[-0.64; -0.05]
Mayo 2019	24	6.9%	-1.26	[-1.79; -0.72]
**Overall**		**51.8%**	**-0.60**	**[-1.04; -0.16]**
Heterogeneity: $Tau^2 = 0.1398$; $Chi^2 = 28.87$, df = 5 ($P < 0.01$); $I^2 = 82.7\%$				
**Underlying liver disease = PBC, PSC, or SSC**				
FITCH	36	9.9%	-0.16	[-0.49; 0.17]
**Underlying liver disease = PFIC**				
MARCH-PFIC	46	10.2%	-0.57	[-0.88; -0.26]
PEDFIC 1	14	6.9%	-0.30	[-0.84; 0.23]
Shneider 2018	12	5.9%	-0.64	[-1.27; -0.02]
**Overall**		**23.0%**	**-0.52**	**[-0.88; -0.16]**
Heterogeneity: $Tau^2 = 0$; $Chi^2 = 0.88$, df = 2 ($P = 0.64$); $I^2 = 0\%$				
**Underlying liver disease = PSC**				
ELMWOOD	23	8.7%	-0.13	[-0.54; 0.29]
**Overall**		**100.0%**	**-0.49**	**[-0.72; -0.27]**
Heterogeneity: $Tau^2 = 0.0801$; $Chi^2 = 36.49$, df = 11 ($P < 0.01$); $I^2 = 69.9\%$				
Test for subgroup differences: $Chi^2 = 8.38$, df = 4 ($P = 0.08$)				

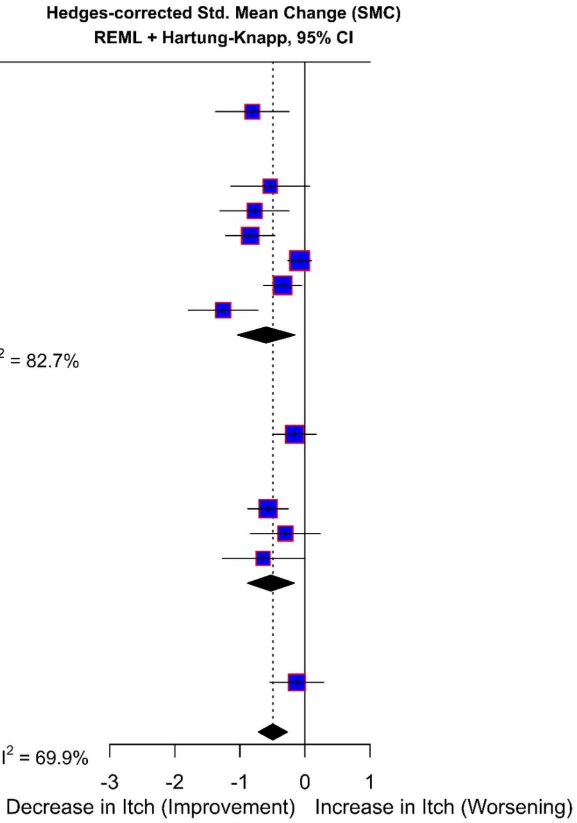

Supplement: Supplementary file 5 [file APT-64-1348-s007.docx]
