# Supplementary material for: Meta‐Analysis: The Placebo Response in Modern Treatments for Cholestatic Pruritus: A Systematic Review and Meta‐Analysis of Randomized Controlled Trials
Source: Aliment Pharmacol Ther. 2026 Sep 24;64(10):1348–61. doi: 10.1111/apt.70915 (PMC13688708; doi:10.1111/apt.70915)
Supplement: Supplementary file 8 [file APT-64-1348-s010.docx]

Risk of bias domains

Study	D1	D2	D3	D4	D5	Overall
ENHANCE	+	+	-	+	+	-
Hirschfield, 2024	+	+	+	+	+	+
GLISTEN	+	+	+	-	+	-
ELATIVE	+	+	+	+	+	+
GLIMMER	+	+	+	+	+	+
Mayo, 2019	+	+	+	+	+	+
ELMWOOD	+	+	+	+	+	+
MARCH-PFIC	+	+	+	+	+	+
ASSERT	+	+	+	+	+	+
Schneider, 2018	+	+	+	+	+	+
PEDFIC 1	+	+	+	+	+	+
FITCH	+	+	+	+	+	+

Domains:
D1: Bias arising from the randomization process.
D2: Bias due to deviations from intended intervention.
D3: Bias due to missing outcome data.
D4: Bias in measurement of the outcome.
D5: Bias in selection of the reported result.

Judgement
- Some concerns
+ Low
