# Supplementary material for: Meta‐Analysis: The Placebo Response in Modern Treatments for Cholestatic Pruritus: A Systematic Review and Meta‐Analysis of Randomized Controlled Trials
Source: Aliment Pharmacol Ther. 2026 Sep 24;64(10):1348–61. doi: 10.1111/apt.70915 (PMC13688708; doi:10.1111/apt.70915)
Supplement: Supplementary file 9 [file APT-64-1348-s011.docx]

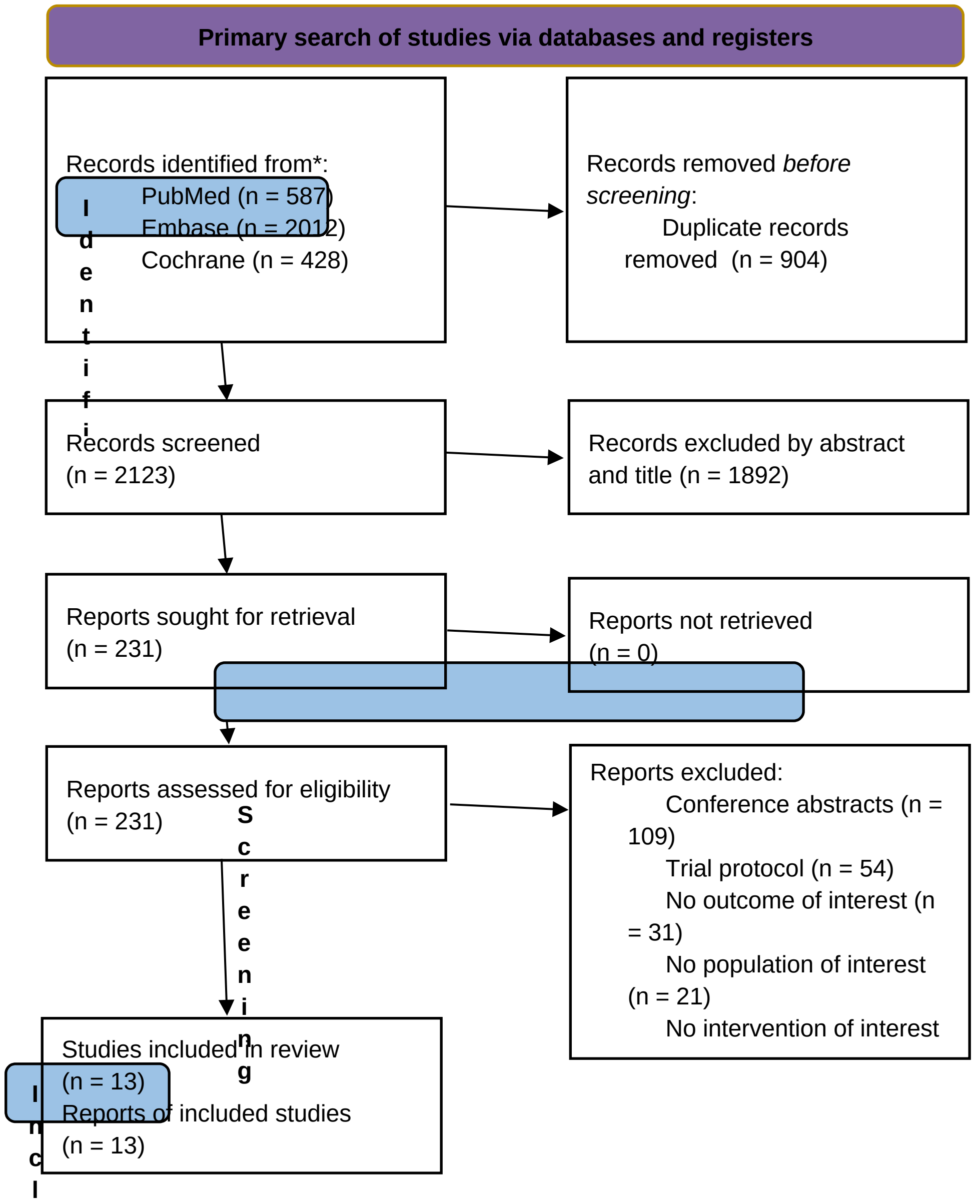
Primary search of studies via databases and registers
Records identified from*:
PubMed (n = 587)
Embase (n = 2012)
Cochrane (n = 428)
Records removed before screening:
Duplicate records removed (n = 904)
Records screened
(n = 2123)
Records excluded by abstract and title (n = 1892)
Reports sought for retrieval
(n = 231)
Reports not retrieved
(n = 0)
Reports assessed for eligibility
(n = 231)
Screening
Reports excluded:
Conference abstracts (n = 109)
Trial protocol (n = 54)
No outcome of interest (n = 31)
No population of interest (n = 21)
No intervention of interest
Studies included in review
(n = 13)
Reports of included studies
(n = 13)
