# Supplementary material for: Meta‐Analysis: The Placebo Response in Modern Treatments for Cholestatic Pruritus: A Systematic Review and Meta‐Analysis of Randomized Controlled Trials
Source: Aliment Pharmacol Ther. 2026 Sep 24;64(10):1348–61. doi: 10.1111/apt.70915 (PMC13688708; doi:10.1111/apt.70915)

Studies	Total (N)	Weight	SMC	95% CI
**Treatment duration = < 5 months**				
ENHANCE	18	8.2%	-0.77	[-1.30; -0.25]
FITCH	36	11.4%	-0.16	[-0.49; 0.17]
GLIMMER	24	9.0%	-0.90	[-1.38; -0.43]
Mayo 2019	24	8.1%	-1.26	[-1.79; -0.72]
Shneider 2018	12	7.0%	-0.64	[-1.27; -0.02]
**Overall**		**43.8%**	**-0.72**	**[-1.24; -0.20]**
Heterogeneity: $Tau^2 = 0.1294$; $Chi^2 = 14.6$, df = 4 ($P < 0.01$); $I^2 = 72.6\%$				
**Treatment duration = ≥ 5 months**				
ASSERT	16	7.7%	-0.81	[-1.37; -0.24]
ELATIVE	12	7.2%	-0.53	[-1.14; 0.07]
GLISTEN	119	13.7%	-0.08	[-0.26; 0.10]
Hirschfield 2024	16	7.9%	-0.75	[-1.31; -0.20]
MARCH-PFIC	46	11.6%	-0.57	[-0.88; -0.26]
PEDFIC 1	14	8.1%	-0.30	[-0.84; 0.23]
**Overall**		**56.2%**	**-0.45**	**[-0.76; -0.14]**
Heterogeneity: $Tau^2 = 0.0588$; $Chi^2 = 14.75$, df = 5 ($P = 0.01$); $I^2 = 66.1\%$				
**Overall**		**100.0%**	**-0.57**	**[-0.81; -0.33]**
Heterogeneity: $Tau^2 = 0.0886$; $Chi^2 = 35.73$, df = 10 ($P < 0.01$); $I^2 = 72.0\%$				
Test for subgroup differences: $Chi^2 = 1.47$, df = 1 ($P = 0.23$)				

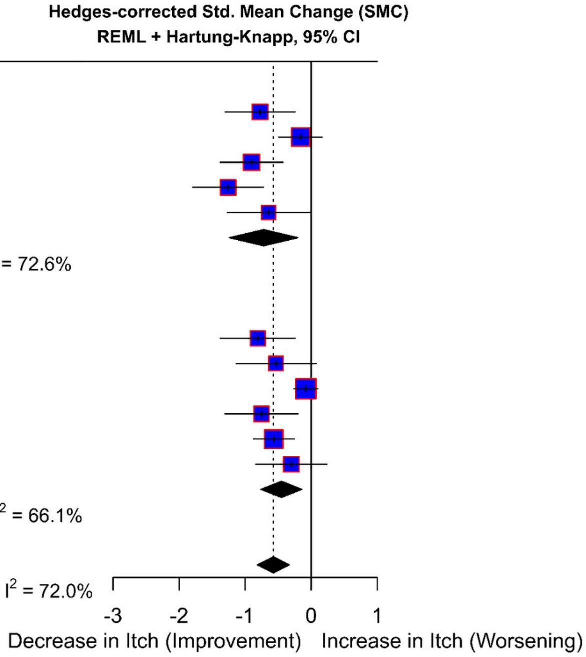

Supplement: Supplementary file 10 [file APT-64-1348-s012.docx]
