# Supplementary material for: Meta‐Analysis: The Placebo Response in Modern Treatments for Cholestatic Pruritus: A Systematic Review and Meta‐Analysis of Randomized Controlled Trials
Source: Aliment Pharmacol Ther. 2026 Sep 24;64(10):1348–61. doi: 10.1111/apt.70915 (PMC13688708; doi:10.1111/apt.70915)

| Studies | Responders | Total | Weight | Proportion | 95% CI |
|---|---|---|---|---|---|
| **Subgroup = Adults** | | | | | |
| ELMWOOD | 0 | 2 | 2.8% | 0.00 | [ 0.00; 84.19] |
| ENHANCE | 3 | 18 | 11.8% | 16.67 | [ 3.58; 41.42] |
| GLISTEN | 51 | 119 | 29.8% | 42.86 | [33.83; 52.25] |
| **Total (95% CI)** | | **139** | **44.4%** | **29.50** | **[ 5.38; 75.50]** |
| Heterogeneity: $Tau^2 = 0.5172$; $Chi^2 = 4.63$, df = 2 (P = 0.10); $I^2 = 57\%$ | | | | | |
| **Subgroup = Children** | | | | | |
| ASSERT | 3 | 16 | 11.6% | 18.75 | [ 4.05; 45.65] |
| MARCH-PFIC | 12 | 46 | 22.5% | 26.09 | [14.27; 41.13] |
| PEDFIC 1 | 2 | 14 | 9.1% | 14.29 | [ 1.78; 42.81] |
| Shneider 2018 | 4 | 12 | 12.4% | 33.33 | [ 9.92; 65.11] |
| **Total (95% CI)** | | **88** | **55.6%** | **24.41** | **[15.21; 36.76]** |
| Heterogeneity: $Tau^2 = 0$; $Chi^2 = 1.6$, df = 3 (P = 0.66); $I^2 = 0\%$ | | | | | |
| **Total (95% CI)** | | **227** | **100.0%** | **27.40** | **[17.93; 39.46]** |
| Heterogeneity: $Tau^2 = 0.2068$; $Chi^2 = 11.73$, df = 6 (P = 0.07); $I^2 = 49\%$ | | | | | |
| Test for subgroup differences: $Chi^2 = 0.27$, df = 1 (P = 0.60) | | | | | |

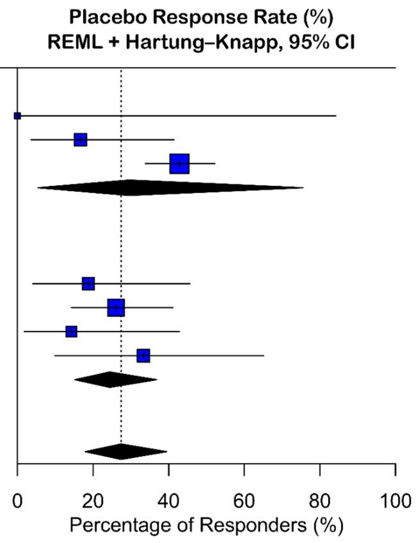

Supplement: Supplementary file 11 [file APT-64-1348-s013.docx]
