# Supplementary material for: Meta‐Analysis: The Placebo Response in Modern Treatments for Cholestatic Pruritus: A Systematic Review and Meta‐Analysis of Randomized Controlled Trials
Source: Aliment Pharmacol Ther. 2026 Sep 24;64(10):1348–61. doi: 10.1111/apt.70915 (PMC13688708; doi:10.1111/apt.70915)

Studies	Placebo Response (5D-Itch Scale) Mean Change	SD	Total	Weight	Pooled Estimate	95% CI
ELATIVE	-1.21	4.00	16	19.5%	-1.21	[-3.17; 0.75]
ENHANCE	-1.42	3.69	19	20.0%	-1.42	[-3.08; 0.23]
FITCH	2.34	4.30	36	20.5%	2.34	[0.94; 3.74]
Hirschfield 2024	-2.16	2.79	15	20.5%	-2.16	[-3.57; -0.75]
Mayo 2019	-6.30	4.74	24	19.6%	-6.30	[-8.20; -4.40]
**Total (95% CI)**			**110**	**100.0%**	**-1.72**	**[-5.54; 2.11]**

Heterogeneity: $Tau^2 = 8.7458$; $Chi^2 = 53.95$, df = 4 ($P < 0.01$); $I^2 = 93\%$

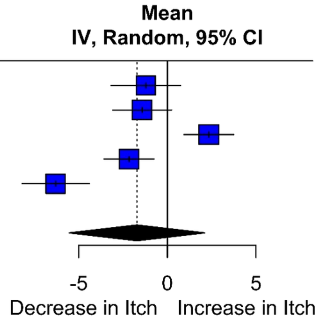

Supplement: Supplementary file 12 [file APT-64-1348-s015.docx]
