# Supplementary material for: Meta‐Analysis: The Placebo Response in Modern Treatments for Cholestatic Pruritus: A Systematic Review and Meta‐Analysis of Randomized Controlled Trials
Source: Aliment Pharmacol Ther. 2026 Sep 24;64(10):1348–61. doi: 10.1111/apt.70915 (PMC13688708; doi:10.1111/apt.70915)

Studies	Total (N)	Weight	SMC	95% CI
**Geographical scope = Multicenter**				
ASSERT	16	7.7%	-0.81	[-1.37; -0.24]
ELATIVE	12	7.2%	-0.53	[-1.14; 0.07]
ENHANCE	18	8.2%	-0.77	[-1.30; -0.25]
FITCH	36	11.4%	-0.16	[-0.49; 0.17]
GLIMMER	24	9.0%	-0.90	[-1.38; -0.43]
GLISTEN	119	13.7%	-0.08	[-0.26; 0.10]
Hirschfield 2024	16	7.9%	-0.75	[-1.31; -0.20]
MARCH-PFIC	46	11.6%	-0.57	[-0.88; -0.26]
Mayo 2019	24	8.1%	-1.26	[-1.79; -0.72]
PEDFIC 1	14	8.1%	-0.30	[-0.84; 0.23]
**Overall**		**93.0%**	**-0.57**	**[-0.84; -0.30]**
Heterogeneity: $Tau^2 = 0.0977$; $Chi^2 = 35.07$, df = 9 ($P < 0.01$); $I^2 = 74.3\%$				
**Geographical scope = USA**				
Shneider 2018	12	7.0%	-0.64	[-1.27; -0.02]
**Overall**		**100.0%**	**-0.57**	**[-0.81; -0.33]**
Heterogeneity: $Tau^2 = 0.0886$; $Chi^2 = 35.73$, df = 10 ($P < 0.01$); $I^2 = 72.0\%$				
Test for subgroup differences: $Chi^2 = 0.05$, df = 1 ($P = 0.83$)				

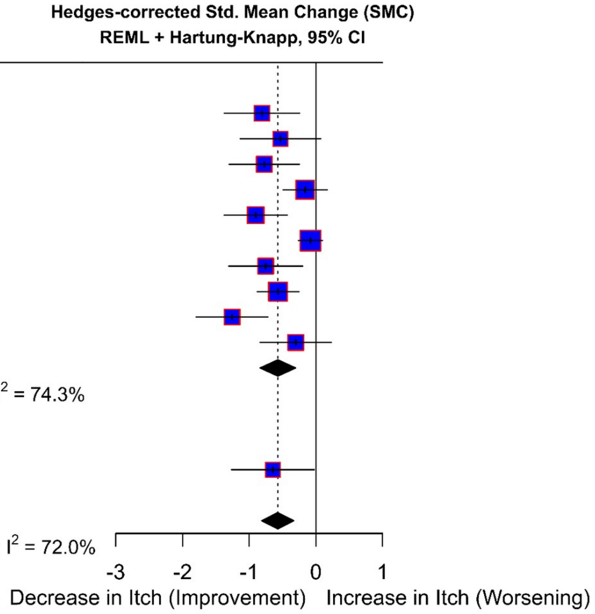

Supplement: Supplementary file 13 [file APT-64-1348-s017.docx]
