# Supplementary material for: Meta‐Analysis: The Placebo Response in Modern Treatments for Cholestatic Pruritus: A Systematic Review and Meta‐Analysis of Randomized Controlled Trials
Source: Aliment Pharmacol Ther. 2026 Sep 24;64(10):1348–61. doi: 10.1111/apt.70915 (PMC13688708; doi:10.1111/apt.70915)

| Studies | Total (N) | Weight | SMC | 95% CI |
|---|---|---|---|---|
| **Geographical scope = Multicenter** | | | | |
| ASSERT | 16 | 6.6% | -0.81 | [-1.37; -0.24] |
| ELATIVE | 12 | 6.1% | -0.53 | [-1.14; 0.07] |
| ELMWOOD | 23 | 8.7% | -0.13 | [-0.54; 0.29] |
| ENHANCE | 18 | 7.0% | -0.77 | [-1.30; -0.25] |
| FITCH | 36 | 9.9% | -0.16 | [-0.49; 0.17] |
| GLIMMER | 36 | 9.1% | -0.84 | [-1.22; -0.46] |
| GLISTEN | 119 | 12.1% | -0.08 | [-0.26; 0.10] |
| Hirschfield 2024 | 48 | 10.5% | -0.34 | [-0.64; -0.05] |
| MARCH-PFIC | 46 | 10.2% | -0.57 | [-0.88; -0.26] |
| Mayo 2019 | 24 | 6.9% | -1.26 | [-1.79; -0.72] |
| PEDFIC 1 | 14 | 6.9% | -0.30 | [-0.84; 0.23] |
| **Overall** | | **94.1%** | **-0.49** | **[-0.73; -0.24]** |
| Heterogeneity: $Tau^2 = 0.0867$; $Chi^2 = 35.7$, df = 10 ($P < 0.01$); $I^2 = 72\%$ | | | | |
| **Geographical scope = USA** | | | | |
| Shneider 2018 | 12 | 5.9% | -0.64 | [-1.27; -0.02] |
| **Overall** | | **100.0%** | **-0.49** | **[-0.72; -0.27]** |
| Heterogeneity: $Tau^2 = 0.0801$; $Chi^2 = 36.49$, df = 11 ($P < 0.01$); $I^2 = 69.9\%$ | | | | |
| Test for subgroup differences: $Chi^2 = 0.22$, df = 1 ($P = 0.64$) | | | | |

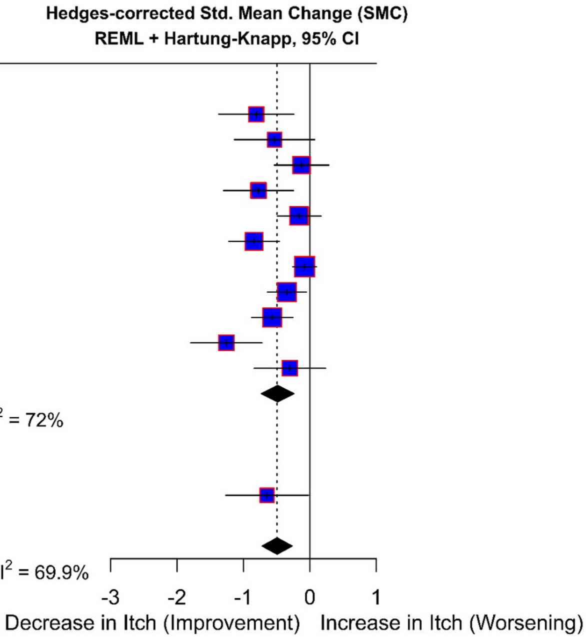

Supplement: Supplementary file 14 [file APT-64-1348-s018.docx]
