# Supplementary material for: Meta‐Analysis: The Placebo Response in Modern Treatments for Cholestatic Pruritus: A Systematic Review and Meta‐Analysis of Randomized Controlled Trials
Source: Aliment Pharmacol Ther. 2026 Sep 24;64(10):1348–61. doi: 10.1111/apt.70915 (PMC13688708; doi:10.1111/apt.70915)

Studies	Placebo Response Mean Change	SD	Total	Weight	Pooled Estimate	95% CI
ELATIVE	-1.25	2.18	12	33.5%	-1.25	[-2.48; -0.01]
ELMWOOD	-0.28	2.18	23	64.3%	-0.28	[-1.17; 0.61]
GLISTEN	-2.15	26.42	119	2.3%	-2.15	[-6.90; 2.60]
**Total (95% CI)**			**154**	**100.0%**	**-0.65**	**[-1.36; 0.07]**

Heterogeneity: $Tau^2 = 0$; $Chi^2 = 1.94$, df = 2 (P = 0.38); $I^2 = 0\%$

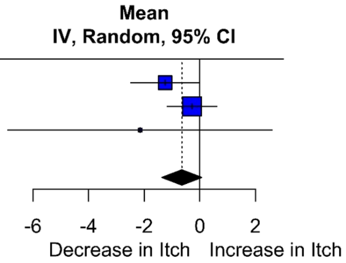

Supplement: Supplementary file 16 [file APT-64-1348-s020.docx]
