# Supplementary material for: Meta‐Analysis: The Placebo Response in Modern Treatments for Cholestatic Pruritus: A Systematic Review and Meta‐Analysis of Randomized Controlled Trials
Source: Aliment Pharmacol Ther. 2026 Sep 24;64(10):1348–61. doi: 10.1111/apt.70915 (PMC13688708; doi:10.1111/apt.70915)
Supplement: Supplementary file 17 [file APT-64-1348-s022.docx]

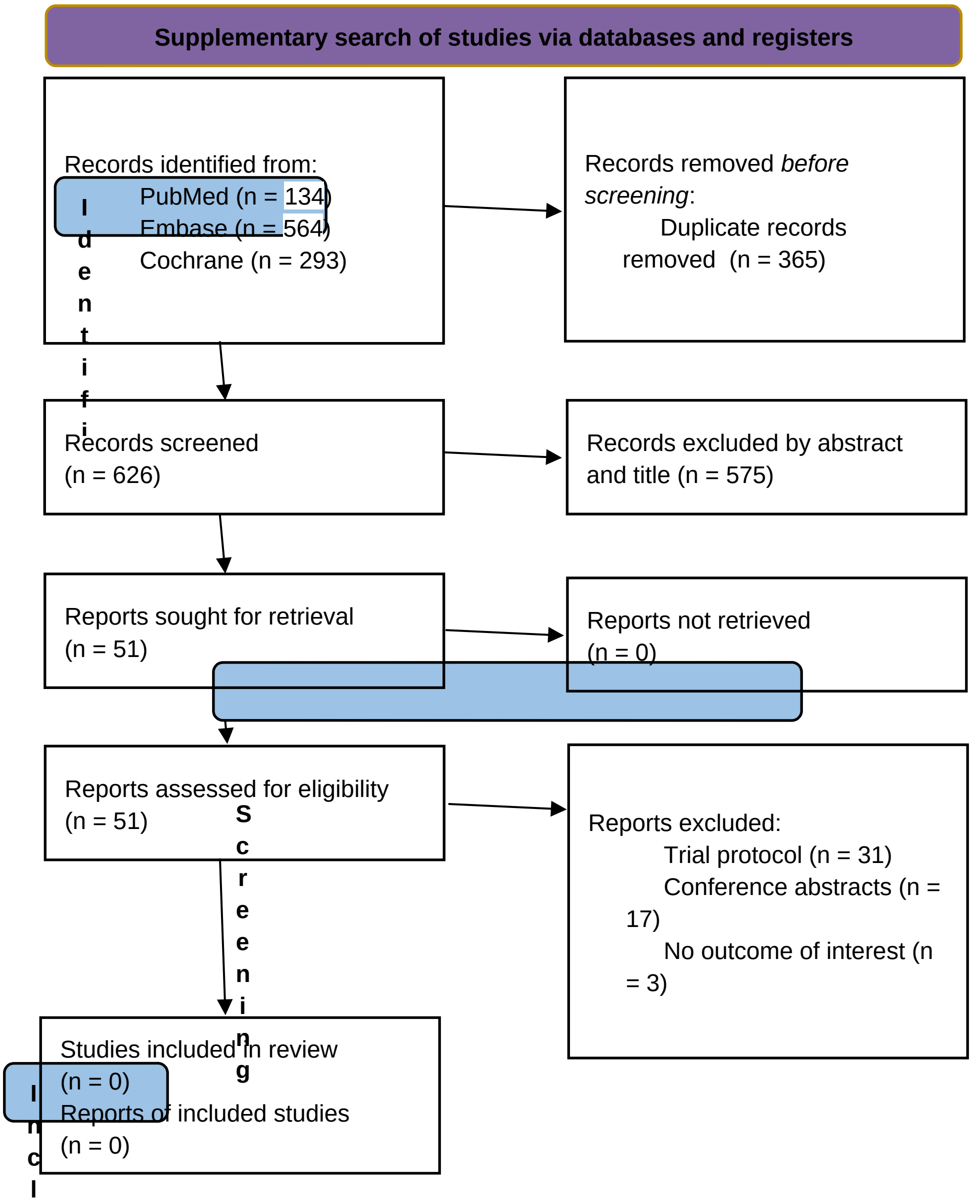
Supplementary search of studies via databases and registers
Records identified from:
PubMed (n = 134)
Embase (n = 564)
Cochrane (n = 293)
Records removed *before screening*:
Duplicate records removed (n = 365)
Records screened
(n = 626)
Records excluded by abstract and title (n = 575)
Reports sought for retrieval
(n = 51)
Reports not retrieved
(n = 0)
Reports assessed for eligibility
(n = 51)
Reports excluded:
Trial protocol (n = 31)
Conference abstracts (n = 17)
No outcome of interest (n = 3)
Studies included in review
(n = 0)
Reports of included studies
(n = 0)
Screening
