# Supplementary material for: Meta‐Analysis: The Placebo Response in Modern Treatments for Cholestatic Pruritus: A Systematic Review and Meta‐Analysis of Randomized Controlled Trials
Source: Aliment Pharmacol Ther. 2026 Sep 24;64(10):1348–61. doi: 10.1111/apt.70915 (PMC13688708; doi:10.1111/apt.70915)

Studies	Total (N)	Weight	SMC	95% CI
**Risk of bias = Low**				
ASSERT	16	6.6%	-0.81	[-1.37; -0.24]
ELATIVE	12	6.1%	-0.53	[-1.14; 0.07]
ELMWOOD	23	8.7%	-0.13	[-0.54; 0.29]
FITCH	36	9.9%	-0.16	[-0.49; 0.17]
GLIMMER	36	9.1%	-0.84	[-1.22; -0.46]
Hirschfield 2024	48	10.5%	-0.34	[-0.64; -0.05]
MARCH-PFIC	46	10.2%	-0.57	[-0.88; -0.26]
Mayo 2019	24	6.9%	-1.26	[-1.79; -0.72]
PEDFIC 1	14	6.9%	-0.30	[-0.84; 0.23]
Shneider 2018	12	5.9%	-0.64	[-1.27; -0.02]
**Overall**		**80.8%**	**-0.53**	**[-0.77; -0.28]**
Heterogeneity: $Tau^2 = 0.0658$; $Chi^2 = 21.18$, df = 9 (P = 0.01); $I^2 = 57.5\%$				
**Risk of bias = Some concerns**				
ENHANCE	18	7.0%	-0.77	[-1.30; -0.25]
GLISTEN	119	12.1%	-0.08	[-0.26; 0.10]
**Overall**		**19.2%**	**-0.38**	**[-4.75; 3.98]**
Heterogeneity: $Tau^2 = 0.1998$; $Chi^2 = 5.96$, df = 1 (P = 0.01); $I^2 = 83.2\%$				
**Overall**		**100.0%**	**-0.49**	**[-0.72; -0.27]**
Heterogeneity: $Tau^2 = 0.0801$; $Chi^2 = 36.49$, df = 11 (P < 0.01); $I^2 = 69.9\%$				
Test for subgroup differences: $Chi^2 = 0.17$, df = 1 (P = 0.68)				

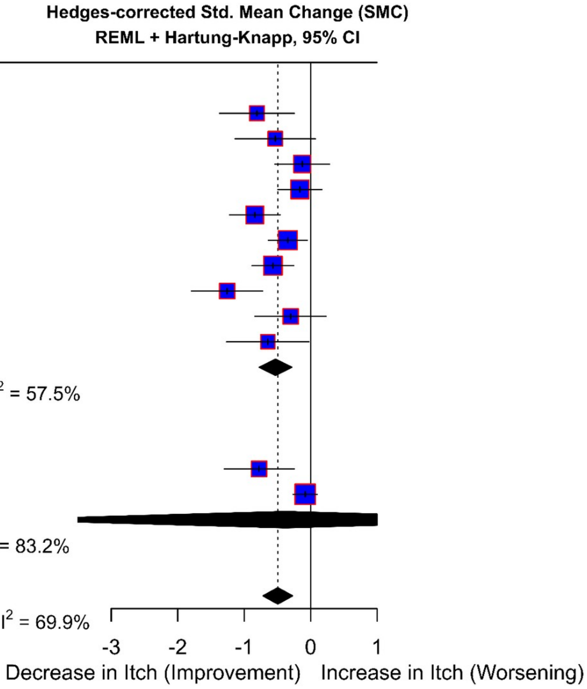

Supplement: Supplementary file 18 [file APT-64-1348-s023.docx]
