# Supplementary material for: Meta‐Analysis: The Placebo Response in Modern Treatments for Cholestatic Pruritus: A Systematic Review and Meta‐Analysis of Randomized Controlled Trials
Source: Aliment Pharmacol Ther. 2026 Sep 24;64(10):1348–61. doi: 10.1111/apt.70915 (PMC13688708; doi:10.1111/apt.70915)

(A)

Placebo Response (Moderate-to-Severe)

Studies	Mean Change	SD	Total	Weight	Pooled Estimate	95% CI
ELATIVE	-0.10	2.61	16	18.9%	-0.10	[-1.38; 1.18]
ENHANCE	-1.75	2.13	16	20.3%	-1.75	[-2.79; -0.70]
GLISTEN	-2.85	3.07	100	22.6%	-2.85	[-3.45; -2.25]
Hirschfield 2024	-1.38	2.75	16	18.5%	-1.38	[-2.73; -0.03]
Mayo 2019	-3.80	2.84	24	19.8%	-3.80	[-4.94; -2.66]
**Total (95% CI)**			**172**	**100.0%**	**-2.02**	**[-3.76; -0.28]**

Heterogeneity: $Tau^2 = 1.6121$; $Chi^2 = 24.05$, df = 4 ($P < 0.01$); $I^2 = 83\%$

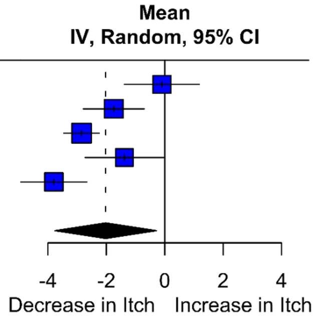

(B)

Placebo Response (Moderate-to-Severe)

Studies	Mean Change	SD	Total	Weight	Pooled Estimate	95% CI
ELATIVE	-2.37	10.74	16	19.1%	-2.37	[-7.63; 2.89]
ENHANCE	-7.18	9.88	16	20.0%	-7.18	[-12.02; -2.34]
GLISTEN	-12.58	12.34	100	24.4%	-12.58	[-15.00; -10.16]
Hirschfield 2024	-7.01	14.71	16	15.4%	-7.01	[-14.22; 0.19]
Mayo 2019	-14.70	10.79	24	21.0%	-14.70	[-19.02; -10.38]
**Total (95% CI)**			**172**	**100.0%**	**-9.13**	**[-15.28; -2.98]**

Heterogeneity: $Tau^2 = 19.1291$; $Chi^2 = 18.27$, df = 4 ($P < 0.01$); $I^2 = 78\%$

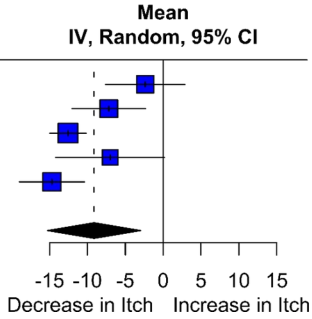

Supplement: Supplementary file 21 [file APT-64-1348-s026.docx]
