# Supplementary material for: Meta‐Analysis: The Placebo Response in Modern Treatments for Cholestatic Pruritus: A Systematic Review and Meta‐Analysis of Randomized Controlled Trials
Source: Aliment Pharmacol Ther. 2026 Sep 24;64(10):1348–61. doi: 10.1111/apt.70915 (PMC13688708; doi:10.1111/apt.70915)
Supplement: Supplementary file 1 [file APT-64-1348-s001.docx]

**Table S5. GRADE assessment of certainty of evidence for analyzed outcomes**

*Certainty ratings apply to pooled within-placebo-arm changes or proportions. They do not represent randomized comparisons between placebo and active treatment and should not be interpreted as certainty in a causal placebo effect.*

| **Certainty assessment** | | | | | | | **Summary of findings** | |
| --- | --- | --- | --- | --- | --- | --- | --- | --- |
| **Participants/** **observations** **(studies)** | **Risk of bias** | **Inconsistency** | **Indirectness** | **Imprecision** | **Publication bias** | **Overall certainty** **of evidence** | **Observed events** | **Pooled estimate** **(95% CI)** |
| **Standardized mean change (SMC) in pruritus intensity in the overall population (follow-up: 0.8 to 13 months; assessed with multiple pruritus instruments, including NRS, WI-NRS, VAS, Adult ItchRO, and pediatric ItchRO/PRUCISION measures)** | | | | | | | | |
| 404 (12 studies) | not serious | not serious^a^ | serious^b^ | not serious | undetected | ⨁⨁⨁◯ **Moderate**^a,b^ | N/A | **SMC -0.49** **(95% CI -0.72 to -0.27)** |
| **Mean change in Worst-Itch Numerical Rating Scale (WI-NRS) among participants with moderate-to-severe pruritus (follow-up: 3 to 13 months; assessed with WI-NRS)** | | | | | | | | |
| 154 (3 studies) | not serious | not serious^a^ | not serious | very serious^c^ | undetected | ⨁⨁◯◯ **Low**^a,c^ | N/A | **MD -0.65** **(95% CI -1.36 to 0.07)** |
| **Mean change in Numerical Rating Scale (NRS) among participants with moderate-to-severe pruritus (follow-up: 3 to 12 months; assessed with NRS)** | | | | | | | | |
| 58 (3 studies) | not serious | not serious^a^ | not serious | very serious^d^ | undetected | ⨁⨁◯◯ **Low**^a,d^ | N/A | **MD -1.62** **(95% CI -2.12 to -1.12)** |
| **Placebo response rate in the overall population (follow-up: 3 to 13 months; assessed using trial-defined clinically meaningful response thresholds)** | | | | | | | | |
| 338 (9 studies) | not serious | not serious^a^ | serious^e^ | serious^f^ | undetected | ⨁⨁◯◯ **Low**^a,e,f^ | 107/338 (31.7%) | **30.0%** **(95% CI 20.6% to 41.4%)** |
| **Mean change in 5-D Itch Scale among participants with moderate-to-severe pruritus (follow-up: 0.8 to 13 months; assessed with 5-D Itch Scale)** | | | | | | | | |
| 110 (5 studies) | not serious | not serious^a^ | not serious | very serious^g^ | undetected | ⨁⨁◯◯ **Low**^a,g^ | N/A | **MD -1.72** **(95% CI -5.54 to 2.11)** |
| **Mean change in PBC-40 itch domain among participants with moderate-to-severe pruritus and primary biliary cholangitis (follow-up: 3 to 13 months; assessed with PBC-40 itch domain)** | | | | | | | | |
| 172 observations (5 studies) | not serious | not serious^a^ | not serious | serious^h^ | undetected | ⨁⨁⨁◯ **Moderate**^a,h^ | N/A | **MD -2.02** **(95% CI -3.76 to -0.28)** |
| **Mean change in PBC-40 overall score among participants with moderate-to-severe pruritus and primary biliary cholangitis (follow-up: 3 to 13 months; assessed with PBC-40 overall score)** | | | | | | | | |
| 172 observations (5 studies) | not serious | not serious^a^ | not serious | serious^i^ | undetected | ⨁⨁⨁◯ **Moderate**^a,i^ | N/A | **MD -9.13** **(95% CI -15.28 to -2.98)** |

**Abbreviations:** CI, confidence interval; GRADE, Grading of Recommendations Assessment, Development and Evaluation; ItchRO, Itch-Reported Outcome; MD, mean difference; N/A, not applicable; NRS, Numerical Rating Scale; PBC, primary biliary cholangitis; PRUCISION, pediatric pruritus and scratching instrument; SMC, standardized mean change based on change scores; VAS, visual analogue scale; WI-NRS, Worst-Itch Numerical Rating Scale.

**Explanations**

**a.** Heterogeneity was evaluated conservatively. For the placebo response rate, I² statistics in proportional meta-analyses can overestimate true data inconsistency. For continuous outcomes, elevated I² was not considered sufficient by itself to justify downgrading because these were descriptive within-placebo-arm syntheses analyzed with random-effects models, while differences in populations, instruments, and reporting sources were considered separately under indirectness where applicable. Therefore, no outcome was downgraded for inconsistency solely on the basis of heterogeneity statistics.

**b.** Downgraded one level for serious indirectness: the SMC combined different cholestatic diseases, adult and pediatric populations, instruments with different constructs and ranges, and both patient- and observer-reported measures.

**c.** Downgraded two levels for very serious imprecision: only 154 participants from three studies contributed, and the 95% CI was wide and crossed no change (-1.36 to 0.07).

**d.** Downgraded two levels for very serious imprecision: only 58 participants from three studies contributed. Although the 95% CI excluded no change, the cumulative sample was extremely small and the estimate was considered fragile.

**e.** Downgraded one level for serious indirectness: adult response was based on a clinically meaningful reduction on 0-10 NRS-based measures, whereas pediatric trials used trial-specific thresholds on 0-4 observer- or patient-reported PRUCISION/ItchRO measures. These definitions and sources of reporting are not fully equivalent.

**f.** Downgraded one level for serious imprecision: the pooled response-rate CI ranged from 20.6% to 41.4%, a width sufficient to materially alter clinical interpretation and trial-design assumptions.

**g.** Downgraded two levels for very serious imprecision: only 110 participants from five studies contributed, and the 95% CI was very wide and crossed no change (-5.54 to 2.11).

**h.** Downgraded one level for serious imprecision: only 172 observations from five studies contributed, and the 95% CI was wide (-3.76 to -0.28), with the lower bound close to no change.

**i.** Downgraded one level for serious imprecision: only 172 observations from five studies contributed, and the 95% CI was wide (-15.28 to -2.98).

General considerations: The source reports were randomized trials, but the estimates synthesized here are non-comparative within-placebo-arm changes or proportions. Consequently, the ratings describe certainty in the pooled descriptive estimates and do not establish a causal placebo effect distinct from natural history, regression to the mean, or other effects of trial participation. Prespecified subgroup analyses were not graded as separate outcomes because they represent subsets of the same bodies of evidence. Under the revised approach, no certainty rating was downgraded for inconsistency solely because of elevated heterogeneity statistics.
