# Supplementary material for: Meta‐Analysis: The Placebo Response in Modern Treatments for Cholestatic Pruritus: A Systematic Review and Meta‐Analysis of Randomized Controlled Trials
Source: Aliment Pharmacol Ther. 2026 Sep 24;64(10):1348–61. doi: 10.1111/apt.70915 (PMC13688708; doi:10.1111/apt.70915)
Supplement: Supplementary file 3 [file APT-64-1348-s003.docx]

| **Study** | **Age** | **Laboratory Criteria** | **Previous Treatment / Other Criteria** | ***Minimum score as inclusion criteria*** | ***Data collection measure*** | ***Description*** | ***Usual recall period*** | ***Assessment frequency*** | ***Mean value calculation*** |
| --- | --- | --- | --- | --- | --- | --- | --- | --- | --- |
| ENHANCE | 18–75 y | ALP ≥1.67× ULN; TB ≤2× ULN | Stable UDCA ≥12 months or UDCA intolerance | *None (Symptomatic: ≥ 4)* | *NRS* | *Simple, self-administered NRS used to measure the intensity of pruritus.* | *Daily* | *0 - 10* | *Baseline scores calculated as the mean of daily recordings; follow-up calculated as weekly means.* |
| Hirschfield 2024 | 18–75 y | ALP ≥1.67× ULN; AST/ALT ≤3× ULN; TB ≤2× ULN; eGFR >45 mL/min/1.73 m²; INR <1.1× ULN; platelets ≥100,000/mm³ | UDCA ≥12 months or intolerance | *None (Symptomatic: ≥ 4)* | *NRS* | *Simple, self-administered NRS used to measure the intensity of pruritus.* | *Daily* | *0 - 10* | *Daily inputs averaged to create weekly means; missing data imputed using mean of adjacent 2 weeks.* |
| GLISTEN | 18–80 y | — | — | *≥ 4 (Monthly score)* | *WI-NRS* | *Simple, self-administered WI-NRS used to measure the peak intensity of pruritus.* | *Twice-daily* | *0 - 10* | *Weekly mean of daily worst scores; monthly score defined as the worst weekly score of the month.* |
| ELATIVE | 18–75 y | ALP ≥1.67× ULN; TB ≤2× ULN | Inadequate response/intolerance to UDCA | *None (Symptomatic: ≥ 4)* | *WI-NRS* | *Simple, self-administered WI-NRS used to measure the peak intensity of pruritus.* | *Daily* | *0 - 10* | *MMRM; intercurrent events treated as missing data under a hypothetical strategy.* |
| GLIMMER | 18–80 y | — | — | *≥ 4 (Monthly score)* | *NRS* | *Simple, self-administered NRS used to measure the intensity of pruritus.* | *Twice-daily* | *0 - 10* | *Daily entries averaged to create weekly means; monthly scores defined as the worst weekly score of the month.* |
| ELMWOOD | 18–75 y | ALP ≥1.5× ULN with <40% variability; TB <2× ULN | Chronic ALP elevation | *None (Symptomatic: ≥ 4)* | *WI-NRS* | *Simple, self-administered WI-NRS used to measure the peak intensity of pruritus.* | *Daily* | *0 - 10* | *ANCOVA model used for analysis; changes from baseline were assessed at week 12.* |
| Mayo 2019 | 18–80 y | — | UDCA ≥6 months (stable dose ≥3 months) or intolerance | *> 4.0 (Average daily score)* | *Adult ItchRO (NRS)* | *Simple, self-administered NRS used to measure the peak intensity of pruritus.* | *Twice-daily* | *0-70* | *Weekly sum score calculated as the sum of daily scores over 7 days; missing daily scores in compliant weeks imputed using the average daily score.* |
| EPICS-I | 18–75 y | ALP ≥1.67× ULN with <30% variability; TB ≤2× ULN | Inadequate response to UDCA ≥12 months | *None specified* | *No unidimensional intensity scales* | *Validated multidimensional questionnaires assessing pruritus burden (no NRS/VAS/WI-NRS used).* | *Varies by item* | *Multi-domain* | *Mean change from baseline at protocol-specific timepoints.* |
| FITCH | ≥18 y | — | — | *≥ 5 (Twice within the week before inclusion)* | *VAS* | *Simple, self-administered, VAS used to measure daily pruritus intensity.* | *Twice-daily* | *0-10* | *Median of all daily scores.* |
| Shneider 2018 | 1–18 y | Genetically confirmed ALGS with cholestasis | — | *≥ 2 (Average daily)* | *ItchRO(Obs)* | *Observer-reported (caregiver) scratching score.* | *Twice-daily* | *0 - 4* | *MMRM; intercurrent events treated as missing data under a hypothetical strategy.* |
| PEDFIC 1 | 0.5–18 y | Serum bile acids ≥100 μmol/L | Genetically confirmed PFIC1/PFIC2 | *≥ 2 (ObsRO or PRO)* | ItchRO (Obs) *and PRO* | *Pruritus and scratching intensity assessed via the PRUCISION instrument. Caregivers used the* ItchRO (Obs) *scale, while patients (aged ≥8 years) used the PRO scale.* | *Twice-daily* | *0 - 4* | *MMRM; intercurrent events treated as missing data under a hypothetical strategy.* |
| ASSERT | Any age | Serum bile acids >ULN | Genetically confirmed ALGS | *≥ 2 (ObsRO or PRO)* | ItchRO (Obs) *and PRO* | *Pruritus and scratching intensity assessed via the PRUCISION instrument. Caregivers used the* ItchRO (Obs) *scale, while patients (aged ≥8 years) used the PRO scale.* | *Twice-daily* | *0 - 4* | *MMRM; intercurrent events treated as missing data under a hypothetical strategy.* |
| MARCH-PFIC | 1–18 y | Biochemical abnormalities and/or pathological evidence of progressive liver disease | — | *≥ 1.5 (Average morning ItchRO)* | *ItchRO(Obs)* | *Observer-reported (caregiver) scratching score.* |  | *0 - 4* | *MMRM; intercurrent events treated as missing data under a hypothetical strategy.* |

**Abbreviations:** ALGS (Alagille Syndrome), ALP (Alkaline Phosphatase), ALT (Alanine Aminotransferase), ANCOVA (Analysis of Covariance), AST (Aspartate Aminotransferase), eGFR (Estimated Glomerular Filtration Rate), INR (International Normalized Ratio), ItchRO (Obs) (Itch-Reported Outcome Observer), MMRM (Mixed Model for Repeated Measures), NRS (Numerical Rating Scale), PBC (Primary Biliary Cholangitis), PFIC (Progressive Familial Intrahepatic Cholestasis), PRO (Patient-Reported Outcome), PSC (Primary Sclerosing Cholangitis), SSC (Secondary Sclerosing Cholangitis), TB (Total Bilirubin), UDCA (Ursodeoxycholic Acid), ULN (Upper Limit of Normal), VAS (Visual Analogue Scale), WI-NRS (Worst-Itch Numerical Rating Scale).
