# Supplementary material for: Meta‐Analysis: The Placebo Response in Modern Treatments for Cholestatic Pruritus: A Systematic Review and Meta‐Analysis of Randomized Controlled Trials
Source: Aliment Pharmacol Ther. 2026 Sep 24;64(10):1348–61. doi: 10.1111/apt.70915 (PMC13688708; doi:10.1111/apt.70915)
Supplement: Supplementary file 14 [file APT-64-1348-s014.docx]

| **Moderator** | **k** | **β (95% CI)** | **SE** | **p-value** | **Residual I² (%)** | **Residual τ²** | **QE** | **pQE** |
| --- | --- | --- | --- | --- | --- | --- | --- | --- |
| **Demographic characteristics** | | | | | | | | |
| Mean age (years) | 11 | −0.00001 (−0.01 to 0.01) | 0.005 | 0.998 | 68.5 | 0.1014 | 32.1 | <0.001 |
| Female participants (%) | 11 | −0.002 (−0.01 to 0.009) | 0.005 | 0.646 | 68.0 | 0.1006 | 34.5 | <0.001 |
| **Clinical parameters** | | | | | | | | |
| Follow-up duration (months) | 11 | 0.0006 (−0.07 to 0.07) | 0.03 | 0.985 | 70.3 | 0.1004 | 35.7 | <0.001 |
| Concurrent UDCA use (participants, %) | 11 | 0.004 (−0.002 to 0.01) | 0.003 | 0.163 | 55.3 | 0.0686 | 20.2 | 0.016 |
| Prior UDCA use (participants, %) | 10 | 0.004 (−0.003 to 0.01) | 0.003 | 0.254 | 57.7 | 0.0755 | 19.0 | 0.015 |
| **Biochemical marker** | | | | | | | | |
| Total bilirubin (mg/dL) | 10 | −0.04 (−0.16 to 0.08) | 0.05 | 0.464 | 57.7 | 0.0608 | 19.6 | 0.012 |

**Notes:** All models were univariable mixed-effects meta-regressions fitted with REML estimation and Knapp–Hartung inference. β represents the change in SMC per one-unit increase in the moderator. Negative SMC values indicate improvement in pruritus.

Baseline NRS was not formally evaluated because it was available from only five studies using a comparable 0–10 NRS-based measure, below the prespecified minimum of 10 studies.

**Abbreviations:** CI, confidence interval; k, number of studies; QE, residual Q statistic; pQE, p-value for QE; R², proportion of between-study heterogeneity explained; SE, standard error; SMC, standardized mean change; UDCA, ursodeoxycholic acid; τ², residual between-study variance.
