# Supplementary material for: Meta‐Analysis: The Placebo Response in Modern Treatments for Cholestatic Pruritus: A Systematic Review and Meta‐Analysis of Randomized Controlled Trials
Source: Aliment Pharmacol Ther. 2026 Sep 24;64(10):1348–61. doi: 10.1111/apt.70915 (PMC13688708; doi:10.1111/apt.70915)
Supplement: Supplementary file 21 [file APT-64-1348-s021.docx]

| Primary search (March 23, 2026) | | |
| --- | --- | --- |
| Database | Search Strategy | Results |
| PubMed | ("Liver Cirrhosis, Biliary"[Mesh] OR "Cholangitis, Sclerosing"[Mesh] OR "PBC"[tiab] OR "primary biliary cholangitis"[tiab] OR "primary biliary cirrhosis"[tiab] OR "PSC"[tiab] OR "primary sclerosing cholangitis"[tiab] OR "idiopathic sclerosing cholangitis"[tiab] OR "secondary sclerosing cholangitis"[tiab] OR "Alagille Syndrome"[Mesh] OR "intrahepatic cholestasis"[tiab] OR "cholestatic liver"[tiab] OR "familial cholestasis"[tiab] OR "inherited cholestasis"[tiab] OR "progressive familial intrahepatic cholestasis"[tiab] OR "PFIC"[tiab] OR "FIC1"[tiab] OR "BRIC"[tiab] OR "BRIC1"[tiab] OR "BRIC2"[tiab] OR "BSEP"[tiab] OR "MDR3"[tiab] OR "Alagille syndrome"[tiab] OR "Alagille-Watson"[tiab] OR "arteriohepatic dysplasia"[tiab])  AND  ("Peroxisome Proliferator-Activated Receptors"[Mesh] OR "Bezafibrate"[Mesh] OR "Fenofibrate"[Mesh] OR "bezafibrate"[tiab] OR "fenofibrate"[tiab] OR “Pemafibrate”[tiab] OR “Ciprofibrate”[tiab] OR "seladelpar"[tiab] OR "elafibranor"[tiab] OR "saroglitazar"[tiab] OR "PPAR agonist*"[tiab] OR "PPAR alpha agonist*"[tiab] OR "PPAR gamma agonist*"[tiab] OR "PPAR delta agonist*"[tiab] OR "PPAR ligand*"[tiab] OR "PPAR modulator*"[tiab] OR "IBAT inhibitor*"[tiab] OR "ileal bile acid transporter inhibitor*"[tiab] OR "ASBT inhibitor*"[tiab] OR "apical sodium-dependent bile acid transporter*"[tiab] OR "maralixibat"[tiab] OR "elobixibat"[tiab] OR "odevixibat"[tiab] OR "linerixibat"[tiab] OR "volixibat"[tiab]) | 587 |
| Embase | ('primary biliary cirrhosis'/exp OR 'sclerosing cholangitis'/exp OR 'alagille syndrome'/exp OR 'pbc':ti,ab,kw OR 'primary biliary cholangitis':ti,ab,kw OR 'primary biliary cirrhosis':ti,ab,kw OR 'psc':ti,ab,kw OR 'primary sclerosing cholangitis':ti,ab,kw OR 'idiopathic sclerosing cholangitis':ti,ab,kw OR 'secondary sclerosing cholangitis':ti,ab,kw OR 'intrahepatic cholestasis':ti,ab,kw OR 'cholestatic liver':ti,ab,kw OR 'familial cholestasis':ti,ab,kw OR 'inherited cholestasis':ti,ab,kw OR 'progressive familial intrahepatic cholestasis':ti,ab,kw OR 'pfic':ti,ab,kw OR 'fic1':ti,ab,kw OR 'bric':ti,ab,kw OR 'bric1':ti,ab,kw OR 'bric2':ti,ab,kw OR 'bsep':ti,ab,kw OR 'mdr3':ti,ab,kw OR 'alagille syndrome':ti,ab,kw OR 'alagille-watson':ti,ab,kw OR 'arteriohepatic dysplasia':ti,ab,kw)  AND  ('peroxisome proliferator activated receptor'/exp OR 'peroxisome proliferator activated receptor agonist'/exp OR 'bezafibrate'/exp OR 'fenofibrate'/exp OR 'bezafibrate':ti,ab,kw OR 'fenofibrate':ti,ab,kw OR 'pemafibrate':ti,ab,kw OR 'ciprofibrate':ti,ab,kw OR 'seladelpar':ti,ab,kw OR 'elafibranor':ti,ab,kw OR 'saroglitazar':ti,ab,kw OR 'ppar agonist*':ti,ab,kw OR 'ppar alpha agonist*':ti,ab,kw OR 'ppar gamma agonist*':ti,ab,kw OR 'ppar delta agonist*':ti,ab,kw OR 'ppar ligand*':ti,ab,kw OR 'ppar modulator*':ti,ab,kw OR 'ibat inhibitor*':ti,ab,kw OR 'ileal bile acid transporter inhibitor*':ti,ab,kw OR 'asbt inhibitor*':ti,ab,kw OR 'apical sodium-dependent bile acid transporter*':ti,ab,kw OR 'maralixibat':ti,ab,kw OR 'shp625':ti,ab,kw OR 'lum001':ti,ab,kw OR 'elobixibat':ti,ab,kw OR 'odevixibat':ti,ab,kw OR 'a4250':ti,ab,kw OR 'linerixibat':ti,ab,kw OR 'gsk2330672':ti,ab,kw OR 'volixibat':ti,ab,kw OR 'shp626':ti,ab,kw) | 2012 |
| Cochrane | ("PBC" OR "primary biliary cholangitis" OR "primary biliary cirrhosis" OR "PSC" OR "primary sclerosing cholangitis" OR "idiopathic sclerosing cholangitis" OR "secondary sclerosing cholangitis" OR "intrahepatic cholestasis" OR "cholestatic liver" OR "familial cholestasis" OR "inherited cholestasis" OR "progressive familial intrahepatic cholestasis" OR "PFIC" OR "FIC1" OR "BRIC" OR "BRIC1" OR "BRIC2" OR "BSEP" OR "MDR3" OR "Alagille syndrome" OR "Alagille-Watson" OR "arteriohepatic dysplasia")  AND  ("bezafibrate" OR "fenofibrate" OR "pemafibrate" OR "ciprofibrate" OR "seladelpar" OR "elafibranor" OR "saroglitazar" OR "PPAR agonist*" OR "PPAR alpha agonist*" OR "PPAR gamma agonist*" OR "PPAR delta agonist*" OR "PPAR ligand*" OR "PPAR modulator*" OR "IBAT inhibitor*" OR "ileal bile acid transporter inhibitor*" OR "ASBT inhibitor*" OR "apical sodium-dependent bile acid transporter*" OR "maralixibat" OR "elobixibat" OR "odevixibat" OR "linerixibat" OR "volixibat") | 428 |
| Supplementary search (July 11, 2026) | | |
| PubMed | ((Cholestasis[MeSH] OR "Cholestasis, Intrahepatic"[MeSH] OR "Cholestasis, Extrahepatic"[MeSH] OR cholesta* OR "biliary cirrhosis" OR "biliary obstruction" OR “biliary stasis” OR "Liver Cirrhosis, Biliary"[Mesh] OR "Cholangitis, Sclerosing"[Mesh] OR "PBC"[tiab] OR "primary biliary cholangitis"[tiab] OR "primary biliary cirrhosis"[tiab] OR "PSC"[tiab] OR "primary sclerosing cholangitis"[tiab] OR "idiopathic sclerosing cholangitis"[tiab] OR "secondary sclerosing cholangitis"[tiab] OR "Alagille Syndrome"[Mesh] OR "intrahepatic cholestasis"[tiab] OR "cholestatic liver"[tiab] OR "familial cholestasis"[tiab] OR "inherited cholestasis"[tiab] OR "progressive familial intrahepatic cholestasis"[tiab] OR "PFIC"[tiab] OR "FIC1"[tiab] OR "BRIC"[tiab] OR "BRIC1"[tiab] OR "BRIC2"[tiab] OR "BSEP"[tiab] OR "MDR3"[tiab] OR "Alagille syndrome"[tiab] OR "Alagille-Watson"[tiab] OR "arteriohepatic dysplasia"[tiab]) AND  (prurit* OR itch* OR scratch* OR prurig* OR Pruritus[MeSH]) AND  (placebo OR Placebo Effect[MeSH]))  NOT  (("Liver Cirrhosis, Biliary"[Mesh] OR "Cholangitis, Sclerosing"[Mesh] OR "PBC"[tiab] OR "primary biliary cholangitis"[tiab] OR "primary biliary cirrhosis"[tiab] OR "PSC"[tiab] OR "primary sclerosing cholangitis"[tiab] OR "idiopathic sclerosing cholangitis"[tiab] OR "secondary sclerosing cholangitis"[tiab] OR "Alagille Syndrome"[Mesh] OR "intrahepatic cholestasis"[tiab] OR "cholestatic liver"[tiab] OR "familial cholestasis"[tiab] OR "inherited cholestasis"[tiab] OR "progressive familial intrahepatic cholestasis"[tiab] OR "PFIC"[tiab] OR "FIC1"[tiab] OR "BRIC"[tiab] OR "BRIC1"[tiab] OR "BRIC2"[tiab] OR "BSEP"[tiab] OR "MDR3"[tiab] OR "Alagille syndrome"[tiab] OR "Alagille-Watson"[tiab] OR "arteriohepatic dysplasia"[tiab]) AND ("Peroxisome Proliferator-Activated Receptors"[Mesh] OR "Bezafibrate"[Mesh] OR "Fenofibrate"[Mesh] OR "bezafibrate"[tiab] OR "fenofibrate"[tiab] OR “Pemafibrate”[tiab] OR “Ciprofibrate”[tiab] OR "seladelpar"[tiab] OR "elafibranor"[tiab] OR "saroglitazar"[tiab] OR "PPAR agonist*"[tiab] OR "PPAR alpha agonist*"[tiab] OR "PPAR gamma agonist*"[tiab] OR "PPAR delta agonist*"[tiab] OR "PPAR ligand*"[tiab] OR "PPAR modulator*"[tiab] OR "IBAT inhibitor*"[tiab] OR "ileal bile acid transporter inhibitor*"[tiab] OR "ASBT inhibitor*"[tiab] OR "apical sodium-dependent bile acid transporter*"[tiab] OR "maralixibat"[tiab] OR "elobixibat"[tiab] OR "odevixibat"[tiab] OR "linerixibat"[tiab] OR "volixibat"[tiab])) | 134 |
| Embase | (( 'cholestasis'/exp OR 'intrahepatic cholestasis'/exp OR 'extrahepatic cholestasis'/exp OR    cholesta* OR 'biliary cirrhosis' OR 'biliary obstruction' OR 'biliary stasis' OR    'biliary liver cirrhosis'/exp OR 'sclerosing cholangitis'/exp OR 'pbc':ti,ab OR    'primary biliary cholangitis':ti,ab OR 'primary biliary cirrhosis':ti,ab OR    'psc':ti,ab OR 'primary sclerosing cholangitis':ti,ab OR    'idiopathic sclerosing cholangitis':ti,ab OR 'secondary sclerosing cholangitis':ti,ab OR    'alagille syndrome'/exp OR 'intrahepatic cholestasis':ti,ab OR    'cholestatic liver':ti,ab OR 'familial cholestasis':ti,ab OR    'inherited cholestasis':ti,ab OR 'progressive familial intrahepatic cholestasis':ti,ab OR    'pfic':ti,ab OR 'fic1':ti,ab OR 'bric':ti,ab OR 'bric1':ti,ab OR    'bric2':ti,ab OR 'bsep':ti,ab OR 'mdr3':ti,ab OR 'alagille syndrome':ti,ab OR    'alagille-watson':ti,ab OR 'arteriohepatic dysplasia':ti,ab)  AND  (prurit* OR itch* OR scratch* OR prurig* OR 'pruritus'/exp)  AND  (placebo OR 'placebo'/exp))  NOT  (('primary biliary cirrhosis'/exp OR 'sclerosing cholangitis'/exp OR 'alagille syndrome'/exp OR 'pbc':ti,ab,kw OR 'primary biliary cholangitis':ti,ab,kw OR 'primary biliary cirrhosis':ti,ab,kw OR 'psc':ti,ab,kw OR 'primary sclerosing cholangitis':ti,ab,kw OR 'idiopathic sclerosing cholangitis':ti,ab,kw OR 'secondary sclerosing cholangitis':ti,ab,kw OR 'intrahepatic cholestasis':ti,ab,kw OR 'cholestatic liver':ti,ab,kw OR 'familial cholestasis':ti,ab,kw OR 'inherited cholestasis':ti,ab,kw OR 'progressive familial intrahepatic cholestasis':ti,ab,kw OR 'pfic':ti,ab,kw OR 'fic1':ti,ab,kw OR 'bric':ti,ab,kw OR 'bric1':ti,ab,kw OR 'bric2':ti,ab,kw OR 'bsep':ti,ab,kw OR 'mdr3':ti,ab,kw OR 'alagille syndrome':ti,ab,kw OR 'alagille-watson':ti,ab,kw OR 'arteriohepatic dysplasia':ti,ab,kw)  AND  ('peroxisome proliferator activated receptor'/exp OR 'peroxisome proliferator activated receptor agonist'/exp OR 'bezafibrate'/exp OR 'fenofibrate'/exp OR 'bezafibrate':ti,ab,kw OR 'fenofibrate':ti,ab,kw OR 'pemafibrate':ti,ab,kw OR 'ciprofibrate':ti,ab,kw OR 'seladelpar':ti,ab,kw OR 'elafibranor':ti,ab,kw OR 'saroglitazar':ti,ab,kw OR 'ppar agonist*':ti,ab,kw OR 'ppar alpha agonist*':ti,ab,kw OR 'ppar gamma agonist*':ti,ab,kw OR 'ppar delta agonist*':ti,ab,kw OR 'ppar ligand*':ti,ab,kw OR 'ppar modulator*':ti,ab,kw OR 'ibat inhibitor*':ti,ab,kw OR 'ileal bile acid transporter inhibitor*':ti,ab,kw OR 'asbt inhibitor*':ti,ab,kw OR 'apical sodium-dependent bile acid transporter*':ti,ab,kw OR 'maralixibat':ti,ab,kw OR 'shp625':ti,ab,kw OR 'lum001':ti,ab,kw OR 'elobixibat':ti,ab,kw OR 'odevixibat':ti,ab,kw OR 'a4250':ti,ab,kw OR 'linerixibat':ti,ab,kw OR 'gsk2330672':ti,ab,kw OR 'volixibat':ti,ab,kw OR 'shp626':ti,ab,kw)) | 564 |
| Cochrane | ((Cholestasis OR "Cholestasis, Intrahepatic" OR "Cholestasis, Extrahepatic" OR cholesta* OR "biliary cirrhosis" OR "biliary obstruction" OR "biliary stasis" OR "Liver Cirrhosis, Biliary" OR "Cholangitis, Sclerosing" OR PBC OR "primary biliary cholangitis" OR "primary biliary cirrhosis" OR PSC OR "primary sclerosing cholangitis" OR "idiopathic sclerosing cholangitis" OR "secondary sclerosing cholangitis" OR "Alagille Syndrome" OR "intrahepatic cholestasis" OR "cholestatic liver" OR "familial cholestasis" OR "inherited cholestasis" OR "progressive familial intrahepatic cholestasis" OR PFIC OR FIC1 OR BRIC OR BRIC1 OR BRIC2 OR BSEP OR MDR3 OR "Alagille syndrome" OR "Alagille-Watson" OR "arteriohepatic dysplasia")  AND  (prurit* OR itch* OR scratch* OR prurig* OR Pruritus)  AND  (placebo OR "Placebo Effect"))  NOT  (("PBC" OR "primary biliary cholangitis" OR "primary biliary cirrhosis" OR "PSC" OR "primary sclerosing cholangitis" OR "idiopathic sclerosing cholangitis" OR "secondary sclerosing cholangitis" OR "intrahepatic cholestasis" OR "cholestatic liver" OR "familial cholestasis" OR "inherited cholestasis" OR "progressive familial intrahepatic cholestasis" OR "PFIC" OR "FIC1" OR "BRIC" OR "BRIC1" OR "BRIC2" OR "BSEP" OR "MDR3" OR "Alagille syndrome" OR "Alagille-Watson" OR "arteriohepatic dysplasia")  AND  ("bezafibrate" OR "fenofibrate" OR "pemafibrate" OR "ciprofibrate" OR "seladelpar" OR "elafibranor" OR "saroglitazar" OR "PPAR agonist*" OR "PPAR alpha agonist*" OR "PPAR gamma agonist*" OR "PPAR delta agonist*" OR "PPAR ligand*" OR "PPAR modulator*" OR "IBAT inhibitor*" OR "ileal bile acid transporter inhibitor*" OR "ASBT inhibitor*" OR "apical sodium-dependent bile acid transporter*" OR "maralixibat" OR "elobixibat" OR "odevixibat" OR "linerixibat" OR "volixibat")) | 293 |
